# Impact of Behavioral Control on the Processing of Nociceptive Stimulation

**DOI:** 10.3389/fphys.2012.00262

**Published:** 2012-08-10

**Authors:** James W. Grau, J. Russell Huie, Sandra M. Garraway, Michelle A. Hook, Eric D. Crown, Kyle M. Baumbauer, Kuan H. Lee, Kevin C. Hoy, Adam R. Ferguson

**Affiliations:** ^1^Cellular and Behavioral Neuroscience, Department of Psychology, Texas A&M UniversityCollege Station, TX, USA; ^2^Brain and Spinal Injury Center, Department of Neurological Surgery, University of California San FranciscoSan Francisco, CA, USA; ^3^Abbott LaboratoriesChicago, IL, USA; ^4^Department of Neurobiology, University of Pittsburgh School of MedicinePittsburgh, PA, USA

**Keywords:** plasticity, instrumental conditioning, learning, spinal cord injury, nociception, BDNF, allodynia, recovery of function

## Abstract

How nociceptive signals are processed within the spinal cord, and whether these signals lead to behavioral signs of neuropathic pain, depends upon their relation to other events and behavior. Our work shows that these relations can have a lasting effect on spinal plasticity, inducing a form of learning that alters the effect of subsequent nociceptive stimuli. The capacity of lower spinal systems to adapt, in the absence of brain input, is examined in spinally transected rats that receive a nociceptive shock to the tibialis anterior muscle of one hind leg. If shock is delivered whenever the leg is extended (controllable stimulation), it induces an increase in flexion duration that minimizes net shock exposure. This learning is not observed in subjects that receive the same amount of shock independent of leg position (uncontrollable stimulation). These two forms of stimulation have a lasting, and divergent, effect on subsequent learning: controllable stimulation enables learning whereas uncontrollable stimulation disables it (learning deficit). Uncontrollable stimulation also enhances mechanical reactivity. We review evidence that training with controllable stimulation engages a brain-derived neurotrophic factor (BDNF)-dependent process that can both prevent and reverse the consequences of uncontrollable shock. We relate these effects to changes in BDNF protein and TrkB signaling. Controllable stimulation is also shown to counter the effects of peripheral inflammation (from intradermal capsaicin). A model is proposed that assumes nociceptive input is gated at an early sensory stage. This gate is sensitive to current environmental relations (between proprioceptive and nociceptive input), allowing stimulation to be classified as controllable or uncontrollable. We further propose that the status of this gate is affected by past experience and that a history of uncontrollable stimulation will promote the development of neuropathic pain.

## Introduction

In the absence of spinal injury, the processing of afferent pain (nociceptive) signals within the spinal cord is regulated by the brain through descending pathways (Sandkühler and Liu, 1998; Gjerstad et al., 2001). In the presence of prolonged nociceptive stimulation, these descending brain pathways can exert a protective effect that dampens neural excitability and, thereby, prevents the sensitization of nociceptive mechanisms (central sensitization) and the development of neuropathic pain (Davies et al., 1983; Faden et al., 1988; Eaton et al., 1997; Hains et al., 2002). Spinal cord injury (SCI) removes this protective effect, allowing spinal systems to react in an unbridled way to on-going afferent input. In the absence of the brain’s oversight, how nociceptive signals impact spinal systems will depend upon intrinsic mechanisms. We will show that these intraspinal systems are tuned to detect whether the nociceptive signal is related to the performance of a particular response (controllable stimulation) and that allowing behavioral control can engage processes that exert a protective/restorative effect that helps to ameliorate the effect of spinal injury. Conversely, a lack of behavioral control can enhance the adverse effect of nociceptive stimulation and promote the development of neuropathic pain, an issue that is discussed in our companion paper (Ferguson et al., under review). Here we focus on the processes that underlie the abstraction of behavioral control and the mechanisms that underlie its long-term benefit.

Because this work relies on concepts developed within the field of learning, we will first provide an overview of some essential concepts in learning and their application to spinal cord plasticity and behavioral rehabilitation. We will then discuss evidence that a nociceptive stimulus has divergent effects depending upon whether it is controllable (response-contingent) or uncontrollable (non-contingent). We will present evidence that a history of behavioral control can reduce the adverse effects of nociceptive stimulation and counter the development of neuropathic pain. We will conclude by reviewing evidence that the beneficial effect of controllable stimulation depends on brain-derived neurotrophic factor (BDNF) and will present a model that integrates these observations. We propose that behavioral control acts to gate how afferent nociceptive signals are processed, and that this determines whether the stimulus has an adaptive or maladaptive effect.

## Learning and Rehabilitation

Our work is guided by an understanding of how systems adapt (i.e., how they learn) in the intact organism. Learning from this perspective represents a form of plasticity, where the effect of a stimulus (*S*), a response (*R*), or an outcome (*O*), depends upon whether the event (the *S*, *R*, or *O*) has previously occurred and its relation to other events (Domjan, 2010). Within this structure, learning is thought of as a process, a mechanism that detects and encodes on-going events and their relation to past experience. Memory represents the preservation of this information over time.

Within this rubric, we use the term outcome to refer to stimulus events that follow a *R*. If instituting a relationship between a particular *R* and an *O* brings about a change in the *R*, the underlying process is sometimes referred to as reinforcement and the *O* a reinforcer. For example, if a rat is placed in a situation wherein pressing a bar yields a food pellet, the bar-press corresponds to the *R* and the food is the *O*. If this contingency brings about an increase in responding, it is commonly said that the presentation of food *reinforced* bar pressing behavior.

A potential source of confusion stems from the fact that an *O* is a stimulus event and, when its stimulus properties (e.g., intensity, duration) are of concern, may be referred to as such. But more often, the term *S* is used to refer to events that signal whether a particular *R*-*O* relation is in effect or to stimulus events that occur irrespective of any particular behavioral *R*. For example, presentation of a *S* alone might bring about a reduction (habituation) or increase (sensitization) in the behavioral *R* elicited by the *S*. Alternatively, interposing a relationship between two stimuli [usually called the conditioned stimulus (CS) and unconditioned stimulus (US)] can bring about a change in the response elicited by the CS [the conditioned response (CR)], a phenomenon known as Pavlovian (classical) conditioning. Finally, a *S* can indicate whether a particular *R*-*O* relation is in effect, in which case the *S* may be referred to as a discriminative stimulus (*S*^D^).

Past work has shown that spinal mechanisms exhibit habituation, sensitization, and are sensitive to CS-US relations (for reviews, see Patterson, 2001; Patterson and Grau, 2001). Here we focus on an alternative form of learning, instrumental conditioning. Learning theorists have traditionally classified behavioral phenomena on the basis of methodology (Grau and Joynes, 2005a). From this view, single stimulus learning (habituation and sensitization) and Pavlovian conditioning depend solely upon the history of stimulus events encountered; a behavioral response may be used as an index of learning, but is not relevant to the environmental relations that produce the learning. In contrast, instrumental learning depends upon the temporal relationship between a behavioral response and an environmental outcome, the *R*-*O* relation (Grau, 2010). For instrumental learning, the response is central – if establishing a contingency between a particular *R* (whether simple or complex) has a lasting, neurally mediated, effect on behavior, the methodology involves instrumental learning. We focus on this form of learning because, from past work, it was not clear whether isolated spinal mechanisms could exhibit this type of learning and because instrumental learning would seem especially relevant to behavioral rehabilitation after SCI.

A key question at this juncture is: why focus on learning? How is this relevant to the recovery of function after SCI? To understand the importance of learning, consider the primary aim of behavioral rehabilitation – to “retrain” the injured system. At its heart, behavioral rehabilitation involves a set of tasks designed to promote the performance of behaviors that will enhance function and the patient’s well being. To the extent that these procedures yield a lasting effect, they involve a form of learning, and to the extent this learning depends on having experienced a particular *R*-*O* relation, they involve instrumental conditioning. The import of these observations is enhanced by the recognition that behavioral rehabilitation remains the most effective treatment for the restoration of function after injury.

Learning will likely also prove essential to medical treatments designed to foster neural growth to bridge an injury, because encouraging axon elongation is only part of the story. Once the injury is spanned, the pattern of synaptic connectivity must be tuned to promote adaptive processes and avoid maladaptive outcomes (e.g., neuropathic pain). Just as experience helps to shape the pattern of connectivity during development, rewiring spinal circuits will require procedures that promote adaptive learning.

## Spinally Mediated Learning

Our claim is that behavioral rehabilitation has a lasting effect because it encourages a form of learning and that this process occurs, in part, within the spinal cord. In subsequent sections, we bolster this claim with physiological and pharmacological studies examining the underlying mechanisms. As we will see, this work suggests that behavioral control may gate nociceptive signals within the dorsal spinal cord and thereby determine whether stimulation has an adaptive or maladaptive effect. But before we get there, we need to reinforce our central claim – that spinal mechanisms can support learning. Addressing past issues has required a detailed behavioral analysis, providing evidence of learning and uncovering some key features of the underlying processes. Indeed, our work turns the usual analysis of instrumental behavior on its head, shifting the focus from the behavioral response (the consequence of learning) to processes related to the sensory cues. Along the way, we will note the implications of this analysis for behavioral rehabilitation and address some issues in terminology that have led to confusion and controversy.

### Habituation, sensitization, and pavlovian conditioning

It is well recognized that spinal systems can exhibit some basic forms of learning (Patterson and Grau, 2001). The focus in these studies has typically been on the functional capacities of the lower (lumbosacral) spinal cord and the central issue is: To what extent can neurons within this region support learning in the absence of input from the brain? To address this issue, researchers typically sever neural communication with the brain by means of a mid-level (thoracic) transection. After this spinal injury, spinally mediated learning can be studied using stimuli applied to the hind limbs or tail. Because nociceptive reflexes remain intact, and provide a means for monitoring the behavioral consequences of stimulation, many studies use stimuli that engage nociceptive fibers. Of course, because ascending sensory fibers have been cut, subjects perceive no pain.

Research using spinally transected animals has established that spinal systems can support single stimulus learning and provided the foundation for the dual-process model of habituation and sensitization (Groves and Thompson, 1970). Though motivated by different concerns, recent work has extended these observations to demonstrate that afferent nociceptive signals can cause a lasting increase in neural excitability within the spinal cord (central sensitization). This sensitization enhances reactivity to tactile stimulation and is thought to contribute to the development of neuropathic pain (Woolf, 1983; Willis, 2001; Latremoliere and Woolf, 2009). The neurochemical systems that support this plasticity have much in common with the machinery that underlies brain-dependent learning and memory within the hippocampus (Sandkühler, 2000; Ji et al., 2003).

There is also considerable evidence that spinal mechanisms are sensitive to *S*–*S* (Pavlovian) relations (Patterson, 2001). In these studies, the stimuli are applied to dermatomes below a complete spinal transection. A common finding (Fitzgerald and Thompson, 1967; Patterson et al., 1973; Beggs et al., 1985; Durkovic, 1986, 2001; Grau et al., 1990; Illich et al., 1994; Joynes and Grau, 1996) is that the physiological/behavioral response elicited by one stimulus (CS) depends upon whether it has been paired with a noxious input (the US) generated using electrical stimulation at an intensity that engages nociceptive fibers. These studies highlight a common feature within this literature – that spinal learning is often studied using nociceptive stimulation/reflexes. For this reason, the work details a form of nociceptive plasticity.

It has been known for decades that spinal mechanisms exhibit single stimulus (a.k.a. non-associative) learning and Pavlovian conditioning (Fitzgerald and Thompson, 1967; Groves and Thompson, 1970). Yet, the initial acceptance of this work was tempered by an intellectual climate that saw “true” learning as associative in nature – as reflecting the *de novo* linking of two arbitrarily paired events. Much has changed in the ensuing years. Researchers found that invertebrates, with neural assemblies far simpler than that found within the spinal cord, also exhibit a range of learning phenomena (Sahley and Crow, 1998). This behavioral work laid the foundation for uncovering the neurobiological mechanisms involved in learning, in both invertebrates and vertebrates (Kandel and Schwartz, 1982; Pittenger and Kandel, 2003). Concurrent studies revealed that learning is often biologically prepared, tuned by the organism’s evolutionary history (Timberlake and Lucas, 1989; Timberlake, 1999). If both a taste and a visual cue (the CSs) are paired with illness (the US), rats acquire an aversion to the taste but not the visual cue (Garcia et al., 1989). If shock is used as the US, these relations are reversed. Recognizing that learning is often prepared is important because demonstrations of learning within the spinal cord, and in invertebrates, routinely take advantage of pre-existing response tendencies. At the same time, our view of what constitutes learning expanded to include non-associative effects (e.g., Domjan, 2010). Indeed, on closer analysis, we now recognize that true associative learning may be the exception, rather than the rule (Grau and Joynes, 2005a,b); in most Pavlovian paradigms, the CS has some capacity to elicit a CR-like response prior to its being paired with the US. Within this broader modern context, evidence of habituation, sensitization, and Pavlovian conditioning demonstrate that spinal systems can learn.

### Instrumental conditioning

**Figure 1:**
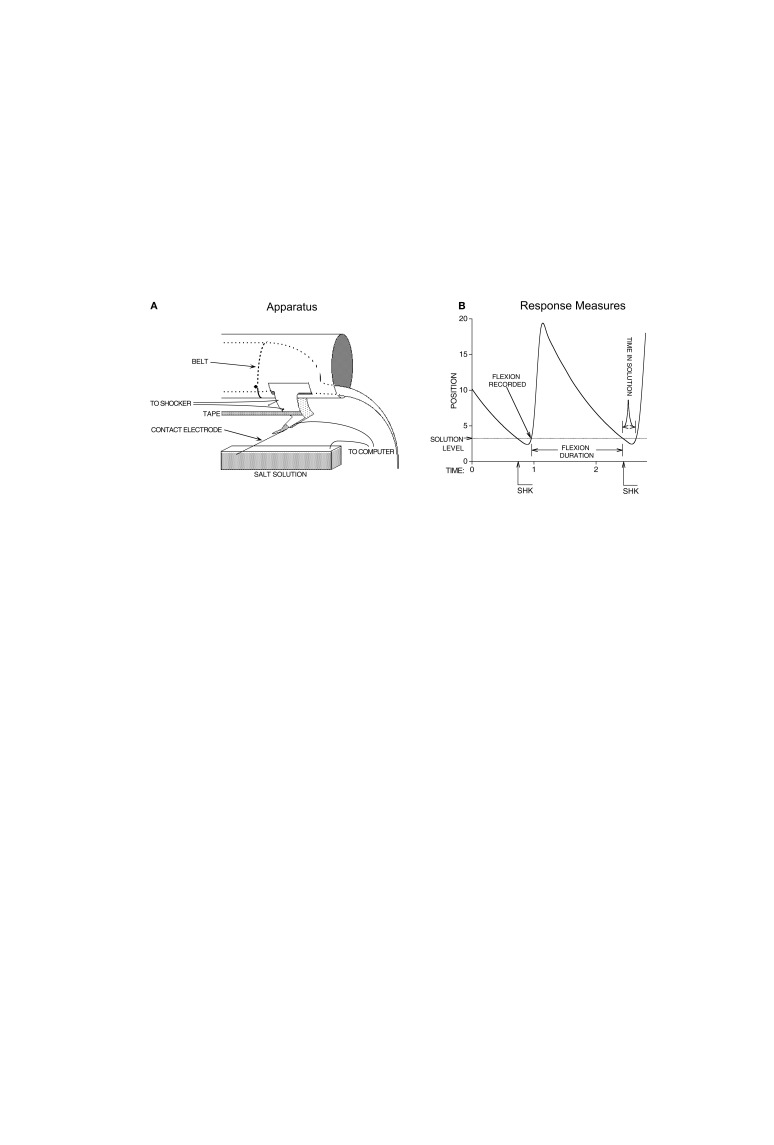
**Apparatus and measures used to study instrumental learning in spinally transected rats**. **(A)** Leg position is monitored by means of a contact electrode that is taped to the rat’s paw. When the electrode touches the underlying salt solution, it completes a circuit that is monitored by a computer. Applying leg-shock elicits a flexion response that lifts the contact electrode and breaks the circuit. **(B)** The response measures derived from leg position over time. Rats given controllable shock receive a shock when the contact electrode falls and touches the underlying solution. This elicits a flexion response that lifts the contact electrode and breaks the circuit, whereupon shock is terminated, the duration of solution contact is recorded, and response number is incremented by one. Yoked animals receive shock independent of leg position and the same criteria are used to monitor time in solution and response number. Adapted from Grau et al. (1998).

What has proven more controversial is whether spinal neurons are sensitive to *R*-*O* (instrumental) relations (discussed in Grau et al., 1998, 2006). To explore this issue, researchers have typically used a variant of the Horridge (1962) procedure. Rats undergo a spinal transection and, after a recovery period, are placed in an opaque tube where they can comfortably rest with their hind limbs hanging freely (Figure 1A). With this apparatus, leg position can be monitored by taping a contact electrode to one hind paw. When the tip of this electrode contacts an underlying salt solution, it completes a circuit, providing a binary measure of whether the leg is extended or flexed. Shock is applied through electrodes that stimulate the tibialis anterior muscle at an intensity that elicits a flexion response. With this apparatus, a *R*-*O* relation can be instituted by administering leg-shock whenever the leg is extended, and terminating shock when the leg is flexed.

To examine whether the *R*-*O* relation matters, researchers often include a second group that receives shock independent of leg position. This is accomplished by experimentally coupling (yoking) a subject that has behavioral control (the master rat) to a second subject (the yoked rat) that receives shock at the same time and for the same duration as the master. For the yoked rat, shock occurs in a non-contingent (uncontrollable) manner.

Using this paradigm, early researchers showed that stimulation of the tibialis anterior muscle yielded different behavioral outcomes in master versus yoked subjects, and from this it was suggested that spinal systems are capable of instrumental conditioning (Buerger and Fennessy, 1970; Buerger and Chopin, 1976; Chopin and Buerger, 1976). This claim was soon challenged (Church and Lerner, 1976; Church, 1989) and, as a result, the standard dogma remained – that instrumental learning requires a brain. In retrospect, the difficulties here stemmed from two sources. The first concerned some methodological issues. The second concerned an over-statement of the results based, in part, on some confusion in terminology (e.g., operant versus instrumental conditioning).

Regarding methodology, some of the issues arose because the research crosses interdisciplinary boundaries. Those performing the studies were generally trained in physiology and neuroscience while the critics were typically trained in experimental psychology and learning theory. Each area naturally brings field-specific concerns regarding the relative importance of different experimental variables. Having demonstrated the basic phenomenon, the physiologists sought to study the underlying neurobiological mechanisms whereas the learning theorists sought a more thorough analysis of the phenomenon. The latter raised concerns regarding group size, experimental controls, non-standardized training regimes, and statistical analyses. While we acknowledge the merit of these criticisms, they can be readily addressed.

More problematic than these methodological issues was the realization that the master-yoke paradigm could generate behavioral differences in the absence of instrumental learning (Church and Lerner, 1976). The difficulty is that a *reactive model*, a mechanical (robotic) system that does not encode the *R*-*O* relation, can produce differential behavior in master and yoked subjects. To see the problem, consider the performance of the yoked rat. If we assume some variability in the rate at which the shocked leg falls, the yoked rat’s leg would reach the underlying solution first roughly half the time. On these trials, the leg will remain extended (touching the solution) until the master rat’s leg is extended, whereupon both subjects receive a shock that elicits a flexion response. Notice that the behavioral contingency effectively drives the master rat’s leg up whenever it is extended, minimizing solution contact relative to the yoked subject and, as a result, a master-yoke difference would emerge in the absence of any learning (for additional details, see Grau et al., 1998, 2006). Because earlier results could be generated by a reactive model, the claim that spinal neurons can support instrumental learning was rejected.

Recognizing these pitfalls, we adopted an alternative measure of learning: flexion duration (Grau et al., 1998). Imagine that, while standing, you experienced a shock to one leg whenever that leg was extended. You would soon learn to maintain your leg in a flexed position, recognizing that, if you allowed it to fall, you would be punished by the presentation of another shock. Likewise, if spinal neurons are sensitive to the *R* (extension)-*O* (shock) relation, subjects should exhibit an increase in flexion duration. In our laboratory, we quantify changes in flexion (response) duration by breaking the 30 min training session into 1 min bins. Within each time bin (i), mean response (flexion) duration is computed for each subject using the following formula:

$$R\text{esponse}\;\text{duratio}{\text{n}}_{i}=\frac{\left(60\;\text{s}-\text{time}\;\text{in}\;\text{solutio}{\text{n}}_{i}\right)}{\text{flexion}\;\text{numbe}{\text{r}}_{i}+1}.$$

Importantly, the reactive model suggested by Church and his colleagues does not anticipate that training with controllable stimulation will lead to an increase in flexion duration (Grau et al., 1998). Indeed, if anything, the higher response rate observed in a mechanical master rat should generate shorter flexion duration scores (relative to the yoked control).

**Figure 2:**
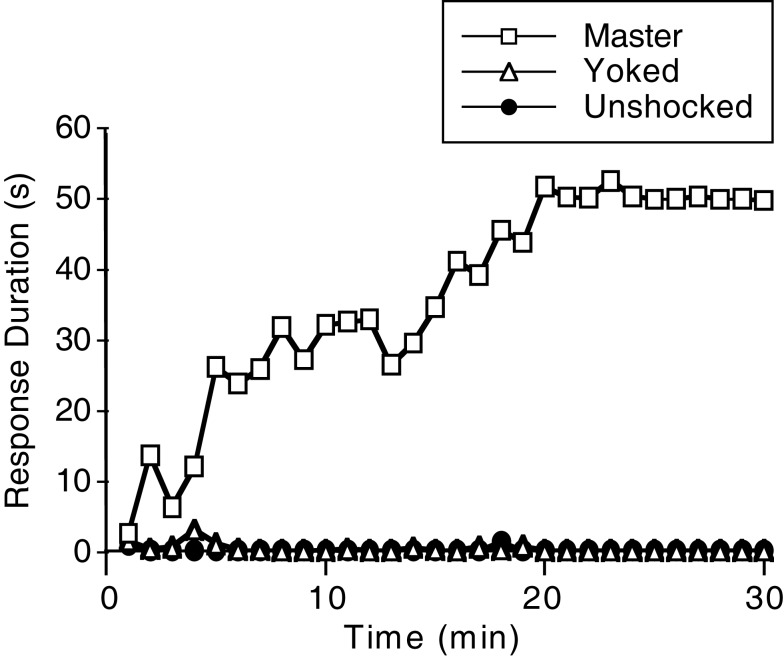
**Impact of training on response duration**. Spinally transected rats received controllable shock (Master), uncontrollable shock (Yoked), or nothing (Unshocked). Master rats exhibited a progressive increase in response (flexion) duration across the 30 min of training. Yoked rats, that received an equal amount of shock independent of limb position, did not exhibit an increase in response duration. Adapted from Grau et al. (1998).

Using response duration as our measure of learning, we examined whether spinal neurons are sensitive to response-outcome relations. Rats underwent a spinal transection and were set-up in the apparatus illustrated in Figure 1A. The behavioral response was monitored as illustrated in Figure 1B. In an effort to standardize the training protocol, and because preliminary data suggested that failures to learn were related to variation in initial flexion force, we adjusted shock intensity to equate flexion force across subjects at the start of training. To address other methodological issues, we standardized other aspects of the training regime (e.g., the duration of training/testing session), used adequate and equal sample sizes, full factorial designs coupled and rigorous statistical techniques. Under these conditions, we found that master, but not yoked, rats exhibited a progressive increase in flexion duration (Figure 2). Interestingly, whether learning was observed depended upon the intensity of the nociceptive stimulation (Grau et al., 1998). If the stimulation was too weak, subjects soon habituated. If the stimulus was very intense, the master rats responded in a mechanical (robotic) manner and generated data that was consistent with a reactive model. These intensity-dependent effects suggest that, in clinical application, training parameters will need to be individually adjusted to an intensity sufficient to maintain behavioral performance without over-stimulating the system.

**Figure 3:**
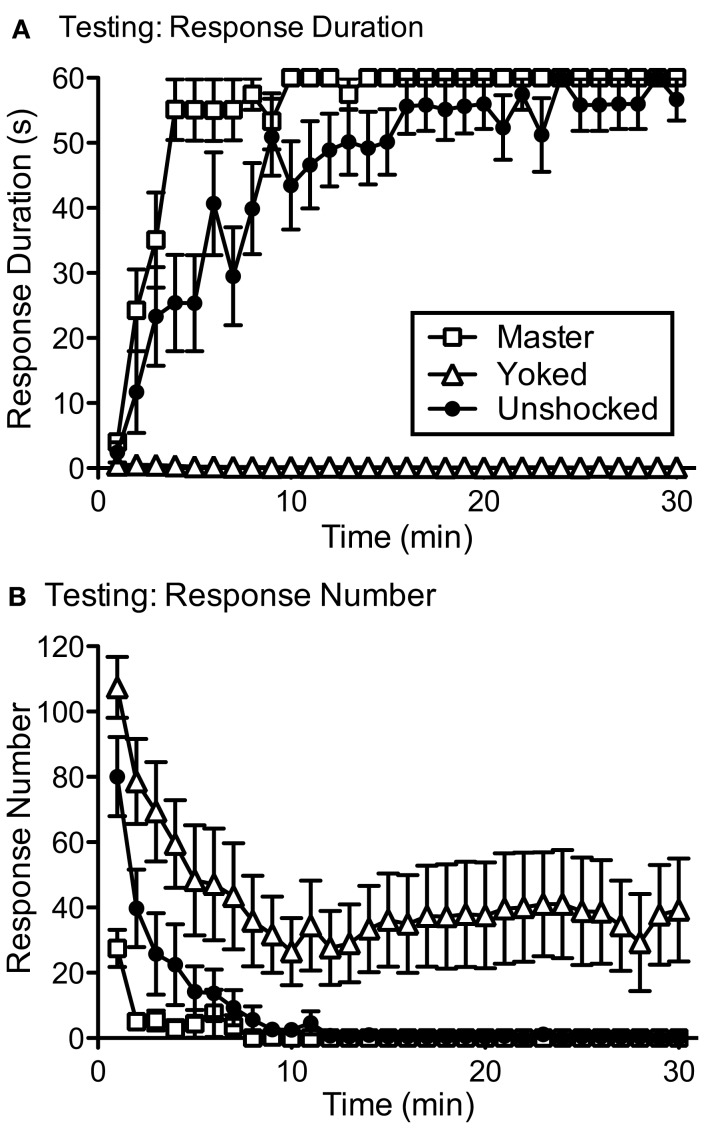
**Testing under common conditions**. **(A)** Subjects that had previously received training with controllable shock (Master), uncontrollable shock (Yoked), or nothing (Unshocked), were tested for 30 min with response-contingent leg-shock. Previously trained rats (Master) exhibited a savings effect that facilitated learning relative to the Unshocked controls. Subjects that had previously received uncontrollable shock (Yoked) did not exhibit an increase in flexion duration (our index of learning) when tested with controllable shock. **(B)** This learning deficit was not due to a failure to respond. Yoked rats exhibited the highest rate of responding and, as a result, repeatedly experienced the response-outcome (*R*-*O*) relation. Adapted from Grau et al. (1998).

The claim of instrumental learning implies a form of memory – that the experience has a lasting effect on behavior. If a behavioral contingency simply drives performance to a particular endpoint, and its effect disappears as soon as the contingency is removed, there is no learning. To demonstrate learning, we must show that the experience has an effect that is preserved over time and is evident when subjects are tested under common conditions (Rescorla, 1988). We addressed this issue by re-equating flexion force [to minimize the contribution of peripheral factors (e.g., muscle fatigue) and single stimulus learning (e.g., habituation)] and re-tested subjects with response-contingent shock (Grau et al., 1998). Previously untreated animals (Unshocked) exhibited a progressive increase in flexion duration when tested with controllable stimulation (Figure 3A). Rats that had undergone training with controllable shock (Master) exhibited some savings and re-acquired the behavior somewhat faster. Surprisingly, rats that previously received uncontrollable shock (Yoked) failed to learn when tested with controllable stimulation, exhibiting a learning deficit reminiscent of the behavioral phenomenon learned helplessness (Maier and Seligman, 1976). Importantly, this learning deficit was not due to a failure to respond. Indeed, rats that had previously received uncontrollable shock exhibited the highest rate of responding (Figure 3B). Thus, yoked rats repeatedly experienced the response-outcome contingency, but failed to exhibit an increase in response duration. It seems that prior exposure to uncontrollable stimulation *disabled* an essential component of the learning process.

### Shock onset reinforces learning: Mechanistic implications

The data presented thus far support the contention that spinal mechanisms are sensitive to *R*-*O* relations. To bolster this conclusion, we sought converging evidence that the *R*-*O* relation matters. According to Church (1964), this issue can be addressed by experimentally manipulating the temporal relationship between the *R* and the *O*. If the *R*-*O* relation matters, then degrading this relationship by inserting a temporal gap should disrupt learning. The implicit assumption here is that learning depends on *R*-*O* contiguity. As we will see, addressing this issue not only uncovers the effective reinforcer, it also informs our model of the underlying process.

**Figure 4:**
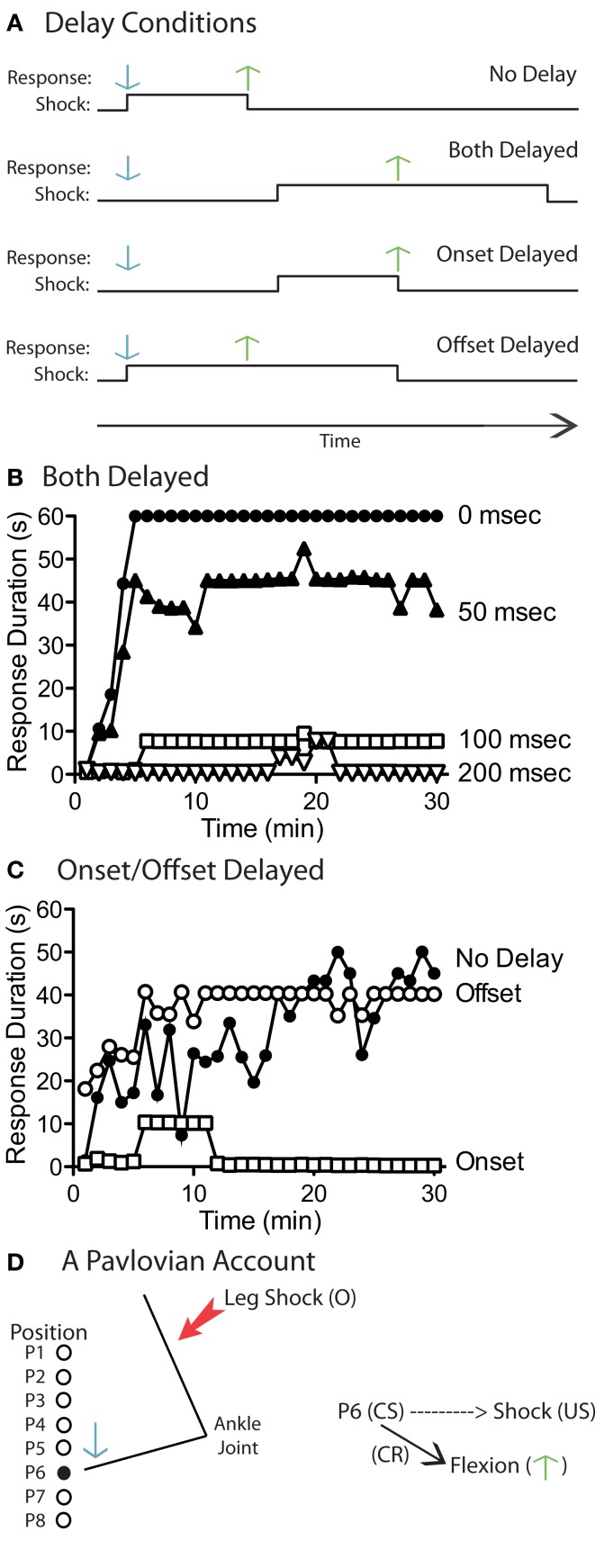
**Relative contribution of shock onset versus offset to learning**. **(A)** Illustration of the manipulations used to explore the impact of delaying shock onset or offset. For each training condition, the onset (up-tick) and offset (down-tick) of shock are indicated over time. It was assumed that, in the absence of a delay (No Delay), shock duration would be approximately 80 ms (Crown et al., 2002b). A downward response (solution contact) and up response are indicated by the down (blue) and up (green) arrows, respectively. The panel illustrates the effect of delaying both onset and offset (Both Delayed), or delaying just onset (Onset Delayed) or offset (Offset Delayed). **(B)** Delaying both shock onset and offset by 100 ms disrupted instrumental learning. **(C)** Delaying onset, but not offset, by 100 ms disrupted learning. **(D)** A theoretical account of the underlying processes. It is assumed that proprioceptive cues (P) provide an afferent signal of limb position. In instrumental training, shock onset (the effective reinforcer) always occurs at the same position (e.g., P6). We suggest that the index of limb position (P6) can function as a Pavlovian conditioned stimulus (CS) and that shock onset may act as an unconditioned stimulus (US). As a result of the CS-US pairing, the CS (P6) may acquire the capacity to elicit a flexion response (the conditioned response, CR). **(B,C)** Adapted from Grau et al. (1998).

To disrupt response-outcome contiguity, we simply delayed both the onset and offset of shock (Grau et al., 1998). For example, for subjects assigned to the 100 ms delay condition, shock did not come on until 100 ms after the contact electrode touched the solution and the shock remained on for an additional 100 ms after the leg was lifted (Figure 4A). Other groups received training with a 0, 50, or 200 ms delay. We found that delaying shock onset and offset by 100 ms or more eliminated learning (Figure 4B).

Next, we examined whether learning was reinforced by shock onset or offset (Grau et al., 1998). Do subjects exhibit an increase in response duration because a downward movement initiates the shock (in behavioral terms, a form of punishment) or because an upward movement turns off the shock (escape) (Domjan, 2010)? To examine these issues, we independently delayed shock onset and offset by 100 ms (Figure 4A). When offset alone was delayed, it had no effect on learning (Figure 4C). When onset was delayed, learning was disrupted. What this suggests is that it is a misnomer to refer to the behavior observed in this paradigm as “escape learning.” Indeed, it is tempting to speculate that escape learning may require more sophisticated (brain-dependent) neural systems.

What we did not fully appreciate when we first described these results is that they have implications regarding the mechanisms that underlie the detection of behavioral control. The findings suggest that the abstraction of behavioral control is linked to events that occur at the onset of the nociceptive stimulus. To see why this is important, consider how the master and yoked rats differ. Only the master rat receives shock when the leg reaches a specific position. The yoked rat receives the same shock, but it occurs independent of leg position. For this difference to matter, spinal systems must register more than shock onset. The system must also have an index of leg position, which we assume is provided by proprioceptive cues. For master rats, shock onset always occurs in the presence of the same cue (leg angle) and our behavioral data suggest that this has special significance – it generates a limb specific increase in flexion duration. Further, for a nociceptive stimulus to have a greater impact when it is given in the presence of a constant proprioceptive cue, the system must have a way of tracking the regularity of this relationship. The system must have a way of encoding (tagging) the leg angle/position at which shock occurred on the previous trial.

An issue that arises at this point is whether the effective code (the index of leg position) is within the animal or built into our apparatus. Have we effectively “tuned” our apparatus (Timberlake and Lucas, 1989), so that all subjects are set-up with a common angle, one that has special biological significance and supports a shock-induced enhancement of flexion duration? Two observations argue against this possibility. First, there is considerable variability across subjects in resting position (i.e., initial foot angle). Second, as we will see later, it is possible to train rats using a different (higher) leg position.

If controllability is tied to the relationship between an index of leg position and shock onset, a lack of control would arise when there is variability in this relationship. For yoked rats, a master generated shock might occur while the yoked rat’s leg is up on one trial and down on the next. Similarly, for master rats, interposing a delay in shock onset would introduce *R*-*O* variability and potentially entrain an inappropriate response (linked to a more extended leg position that maintains solution contact).

These observations have important implications for how we characterize the mechanisms that underlie instrumental learning. While it is natural to assume that *R*-*O* learning involves a motoric effect, our analysis suggests that much of the work may be accomplished on the sensory side – that behavioral control is registered, based on the proprioceptive context in which the stimulus occurs. If that context is constant, the stimulus is encoded as controllable. If it varies, the stimulus is encoded as uncontrollable. From this view, early sensory systems may allow us to directly perceive whether or not a stimulus is response-contingent or non-contingent.

In introducing a cue (proprioceptive feedback), we open the door to a seemingly new account of how spinal systems could support instrumental learning. A signal indicative of leg position could act like a Pavlovian CS which, when paired with the onset of a nociceptive stimulus, acquires the capacity to drive a flexion response (the CR; Figure 4D). From this view, after a shock-elicited flexion is generated, the leg will begin to fall back to a relaxed position. As the ankle approaches the angle at which shock occurs, proprioceptive cues drive a motor response (flexion) that slows the rate of descent, yielding an increase in response duration. Of course, we are not the first to suggest that Pavlovian conditioning may contribute to instrumental learning. Indeed, decades ago Konorski recognized that Pavlovian mechanisms could contribute to instrumental behavior in a flexion paradigm (Konorski and Miller, 1937; Konorski, 1948).

As a result of instrumental training, an active behavioral response (increased flexion) is established. As we will see below, we have established that intraspinal mechanisms mediate the process of learning. We have not, however, specified how this process produces an increase in flexion duration (the memory); it could reflect an intraspinal modification of motor neuron activity within the ventral horn or a selective enhancement of the efferent output. Nor do we know what constitutes the presumed proprioceptive signal; it could be mediated by an index of the static angle or a vector that describes a movement toward that angle. In either case, our results suggest that a passive leg movement is sufficient to generate the requisite signal, because an external force (gravity) draws the leg downward. From the subject’s perspective, it should not matter whether the leg was moved by gravity, the experimenter (or therapist), or a mechanical device – all that should matter is that the onset of the nociceptive stimulus is regularly paired with movement toward a particular leg position. This suggests that, within the clinic, new instrumental behavior could be established through a form of guided therapy, wherein movement of the patient’s limb is regularly paired with the onset of biologically significant (nociceptive) cue. Our work suggests that the success of training will be modulated by temporal regularity (i.e., strong response-outcome contiguity), the extent to which the learning is biologically prepared, and whether prior experience has engaged an intraspinal system that opposes (disables) new learning.

We have suggested that a form of Pavlovian conditioning contributes to instrumental behavior, and in so doing, have seemingly blurred the distinction between these two forms of learning. Indeed, the reader may wonder, if common mechanisms are at work, why maintain separate terms? Here, and elsewhere, it is clear that biological systems often rely on common elements to subserve distinct functions. While this commonality simplifies our analysis, more molar (behaviorally relevant) descriptions of how the system operates retain explanatory value. At the level relevant to behavioral rehabilitation, only instrumental conditioning depends on the relationship between a particular response and an outcome. The fact that *R*-*O* and *S*–*S* relations may be encoded using similar biological machinery simplifies our analysis and may suggest novel treatments. But from the experimenter’s and patient’s perspective, the triggering events differ (a behavioral *R* versus an external CS), and for this reason, the distinction still holds sway.

### Relation to operant behavior and passive avoidance (punishment)

There is another theoretical implication of our analysis of spinal learning that speaks to an earlier issue and criticisms of this line of work. As noted above, the idea that spinal mechanisms can support instrumental learning has been challenged. Yet, if given the mechanistic account provided above, we expect few would question the claim. Why such a disconnect? At the heart of the problem, we believe, was a casualness in the use of terms that mistakenly implied a form of over-generalization. To see this, it is useful to consider Skinner’s (1938) distinction between respondent and operant behavior. Skinner suggested that respondent behavior is “elicited” (reflexive in nature) whereas operant responses are “emitted.” In the latter case, the organism could operate on its environment in many ways and performance may be affected by a variety of reinforcers. Ideally, such behavior is relatively unprepared and flexible. To the extent that this is true, we can arbitrarily decide to train any one of a range of responses using a variety of reinforcers. On these criteria, spinal learning will likely fail. We cannot arbitrarily train an extension or flexion using the same outcome. Nor can we train a given behavior using a variety of reinforcers. These limits arise because spinal learning occurs within a highly prepared system, in which the outcome elicits a defined response and our theoretical account evokes the language of Pavlovian conditioning. In Skinner’s terminology, this represents a form of respondent conditioning. We mention this because the terms instrumental conditioning and operant learning are sometimes used as synonyms. For both, performance depends on the *R*-*O* contingency, but the historical roots (and presumed mechanisms) differ. While the term operant was coined by Skinner to describe emitted behavior, the term instrumental conditioning has its roots in the reflexive tradition of Thorndike and Hull (Hillgard and Marquis, 1940), who assumed reinforcers act by modifying *S*-*R* reflexes. What is important here is that the term instrumental conditioning includes examples of learning that involve a modification of reflexive behavior, which Skinner would classify as a kind of respondent. The implication is that instrumental conditioning represents a broader term, that includes cases of *R*-*O* learning that are biologically prepared (based on pre-existing reflexes) as well as instances that are relatively unprepared. From this view, the term operant behavior refers to a subcategory of instrumental conditioning and is best applied to examples that seem non-respondent (non-reflexive) in nature (see Grau, 2010). Because spinally mediated instrumental conditioning involves the modification of a pre-existing reflex, it would not (from our view) constitute an example of operant behavior (Grau et al., 1998, 2006).

Various forms of instrumental conditioning can be classified depending upon the nature of the *O* (appetitive versus aversive) and whether the behavioral response causes the *O* to occur or be omitted (Domjan, 2010). Above, we showed that the effective *O* in our spinal preparation is shock onset. In behavioral terms, this suggests that learning occurs because the initiating response (a leg extension) is followed by shock, a form of punishment. Punishment is a kind of *passive avoidance*, in which the onset of a nociceptive stimulus brings about a decrease in a behavioral response (the leg extending). In behavioral terms, this seems true. But the description misses the fact that this learning must involve more than an inhibition of a behavior (extension). It must also involve an active process, in which an increase in flexion magnitude reduces net shock exposure. This view mirrors a popular account of punishment in intact subjects (Estes, 1944, 1969). Consider a common paradigm in which rats are placed in a two-sided chamber, with one side brightly lit while the other side is painted black and dimly illuminated. Whenever the rat enters the dark side, it receives a shock. Subjects soon learn not to enter the dark chamber and, in behavioral terms, this reflects a kind of passive avoidance. However, at a mechanistic level, an active process is likely at work. Rats innately prefer the dark side of a chamber and, as a result, have a tendency to enter that context. The dark context (the CS) is then paired with shock (the US), establishing a conditioned fear to the shocked environment that acts like an invisible fence to repel the subject (Domjan, 2010). Here too, what appears to reflect a behaviorally passive process (avoiding the shocked chamber) is maintained by an active, stimulus-elicited, physiological response (conditioned fear elicited by the shocked context).

In summary, we have shown that spinal neurons are sensitive to a *R*-*O* relation and provided evidence that this learning involves, at a behavioral level, a form of passive avoidance (punishment) in which the onset of a nociceptive cue reduces the probability of a specific response (leg extension). At a mechanistic level, we suggest that this process reflects the development of an active process in which an afferent signal indicative of leg position acquires the capacity to drive a flexion response. We assume that the system is built to quickly detect such relations and, in this way, is biased (biologically prepared) in favor of detecting control. Registering the relationship between an index of position/movement and external stimulation would allow the organism to, in a sense, directly perceive control. This position is analogous to a Gibsonian account of depth perception (Gibson, 1979), which showed how information available within the two-dimensional signal detected by the retina (e.g., texture gradients) could provide a cue for depth. In many cases, no down-stream processing (e.g., the computation of binocular disparity) is needed – depth can be directly perceived. Likewise, our account ties the detection of behavioral control to sensory, rather than motor, systems. In terms of spinal anatomy, our analysis suggests a shift in focus, from the ventral to the dorsal horn.

## Uncontrollable Stimulation

The present review focuses on the consequences of controllable stimulation – how behavioral control is detected and how it affects spinal plasticity. A lack of behavioral control could theoretically have no effect, beyond the unconditioned consequences of stimulation *per se*. Our results suggest otherwise, that uncontrollable stimulation engages an active cellular process that has a lasting effect on spinal plasticity. This process is not neutral with respect to instrumental learning, but instead, actively opposes it. The mechanisms that underlie this inhibitory effect are discussed in detail in our companion article (Ferguson et al., under review; also see Baumbauer et al., 2009b). Here, we provide a short overview focusing on concepts relevant to the interaction between controllable and uncontrollable stimulation.

To study the consequences of uncontrollable stimulation, and the underlying neurobiological mechanisms, Crown et al. (2002b) simplified our paradigm by developing a computer program that emulated the variable shock sequence produced by a typical master rat during the first 5–10 min of training. This program generates brief (80 ms) shocks that occur at a variable interval with an average inter-stimulus interval (ISI) of 2 s. Using this program, Crown et al. (2002b) showed that just 6 min of stimulation (approximately 180 shocks) inhibits instrumental learning for up to 48 h. Additional studies showed that the induction of this effect requires protein synthesis (Patton et al., 2004; Baumbauer et al., 2006).

Interestingly, uncontrollable intermittent shock to one hind leg inhibits learning independent of whether subjects are tested on the same (ipsilateral) or opposite (contralateral) leg (Joynes et al., 2003). Indeed, uncontrollable intermittent shock applied to the tail is just as effective (Crown et al., 2002b). These observations suggest that a common system, within the spinal cord, underlies the induction and maintenance of the learning deficit. Further evidence for spinal mediation was obtained by cutting the sciatic nerve prior to intermittent leg-shock (Joynes et al., 2003). When sensory transmission was disrupted in this manner, leg-shock had no effect on learning when subjects were tested on the contralateral leg. Likewise, inactivating the spinal cord (using the Na^+^ channel blocker lidocaine) prior to intermittent shock blocks the induction of the deficit. The induction of the deficit can also be blocked by the spinal application [an intrathecal (i.t.) injection] of an NMDA receptor (NMDAR) antagonist (MK-801), a mGluR1 antagonist (CPCCOEt), or a GABA_A_-R antagonist (bicuculline; Joynes et al., 2004; Ferguson et al., 2006, 2008). Non-neuronal systems (glia and cytokines) also contribute to the induction of the learning deficit (Young et al., 2007; Vichaya et al., 2009; Huie et al., 2012a). The expression of the deficit is blocked by both bicuculline and pretreatment with an opioid antagonist (naltrexone or nor-BNI; Ferguson et al., 2003; Joynes and Grau, 2004; Washburn et al., 2008).

The fact that an opioid antagonist given prior to testing blocks the expression of the learning deficit led us to hypothesize that intermittent shock might inhibit learning because it induces a lasting, opioid-dependent, inhibition of nociceptive processing (antinociception). Indeed, we had previously shown that a long-continuous tail-shock can induce a robust antinociception [inferred from the inhibition of tail-withdrawal from a noxious thermal stimulus (the tail-flick test)] (Meagher et al., 1993). To explore this possibility, Crown et al. (2002b) examined whether exposure to 6 min of intermittent shock induces antinociception. As a positive control, other spinally transected rats received 6 min of continuous tail-shock. Continuous shock induced a robust antinoception, but intermittent shock had no effect. Moreover, when we then tested the capacity for instrumental learning, we found that only intermittent stimulation impaired learning. Continuous shock to the tail not only failed to induce a deficit, it exerted a protective effect that prevented the induction of the learning deficit by intermittent leg-shock (Crown et al., 2002b).

To further explore how intermittent shock affects behavioral reactivity, Ferguson et al. (2006) assessed responsiveness to mechanical stimulation (von Frey stimuli) applied to the mid-plantar surface of the hind paw. We found that intermittent shock enhanced mechanical reactivity (EMR). EMR is of clinical interest because it is generally assumed that the sensitization of nociceptive circuits within the spinal cord affects *both* motor reactivity and the signal relayed to the brain, causing a previously innocuous stimulus to be “perceived” as painful (the clinical definition of allodynia). While this working model has proven valuable, it must be remembered that it is based on an assumed relation and that further work will be needed to determine whether manipulations that affect motor reactivity within an animal model have a parallel effect on human pain. For these reasons, when describing an increase in motor reactivity to tactile stimulation in spinally transected rats, we will refer to it in behavioral terms as EMR.

**Figure 5:**
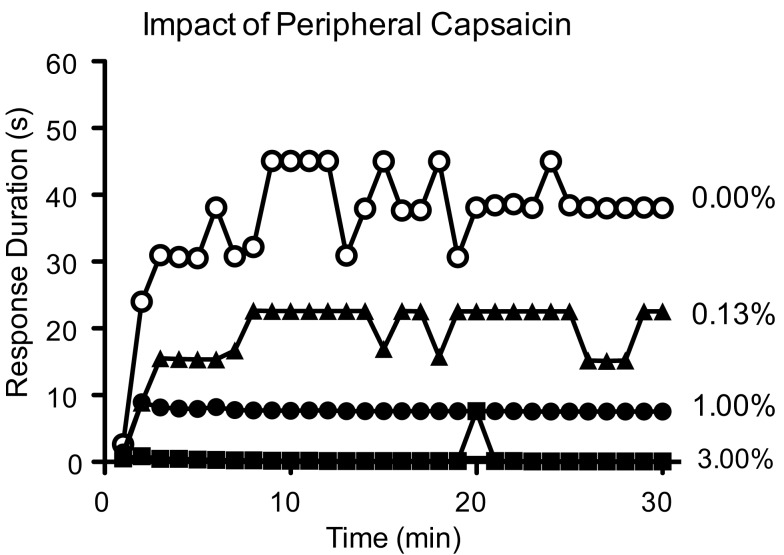
**Peripheral inflammation from capsaicin treatment induces a dose-dependent disruption in instrumental learning**. Adapted from Hook et al. (2008).

EMR is often observed after treatments that induce peripheral inflammation (e.g., intradermal application of formalin or capsaicin) and has been linked to a lasting NMDAR-dependent increase in neural excitability within the spinal cord (central sensitization) and the development of neuropathic pain (Woolf and Thompson, 1991; Coderre et al., 1993; Herrero et al., 2000). Perhaps intermittent, uncontrollable, shock induces a similar effect. If so, this could also explain the disruption in instrumental learning. Within the hippocampus, diffusely saturating NMDAR-dependent plasticity can block the induction of long-term potentiation (LTP) within a selective pathway (Moser and Moser, 1999). Likewise, inducing central sensitization could saturate NMDAR-mediated plasticity within the spinal cord and thereby disrupt the acquisition of selective response modifications (instrumental learning). If this hypothesis is true, then treatments that produce central sensitization should inhibit instrumental learning. Supporting this, Hook et al. (2008) showed that peripheral application of capsaicin produces a dose-dependent inhibition of instrumental learning (Figure 5).

In the uninjured state, descending systems normally exert a protective effect that inhibits the induction of the learning deficit (Crown and Grau, 2005). Supporting this, if intermittent shock is given prior to a spinal transection, it has no effect on spinal learning. This protective effect appears to depend on serotonergic fibers that descend through the dorsolateral funiculus (DLF). Crown and Grau (2005) also demonstrated that bilateral lesions limited to the DLF remove the brain-dependent protective effect. So too does i.t. application of the serotonin 5-HT_1A_ antagonist (WAY 100635). Conversely, spinally transected animals given 5-HT, or the 5-HT_1A_ agonist 8-OH DPAT, prior to intermittent shock do not develop a learning deficit.

Interestingly, this brain-dependent protective effect is not observed in anesthetized rats. Supporting this, intact rats given intermittent tail-shock while anesthetized with pentobarbital, and then transected, exhibit a learning deficit (Washburn et al., 2007). This suggests that noxious stimulation during surgery can adversely affect spinal systems, to inhibit adaptive plasticity and potentially promote the development of neuropathic pain.

In summary, we have shown that intermittent uncontrollable shock induces a lasting inhibition of instrumental learning. This deficit involves a NMDAR-dependent form of plasticity that may be related to the induction of a central sensitization-like process. Spinal injury allows this maladaptive process to develop by releasing lower neural systems from a brain-dependent process that counters the consequences of uncontrollable nociceptive stimulation, possibly by dampening the development of over-excitation and the induction of central sensitization.

## Controllable Stimulation

Returning to the focus of the present paper, we will present evidence that controllable stimulation engages a spinally mediated process that has a protective/restorative effect and provide evidence that this process depends on the neurotrophin BDNF.

### Behavioral properties

Recognizing that peripheral changes could contribute to instrumental performance, we first sought evidence that the change in flexion duration (our index of learning) depended upon spinal neurons. Again, we assessed the impact of disrupting the afferent signal (by cutting the sciatic nerve) and inactivating the cord (through i.t. application of lidocaine). After both manipulations, subjects failed to learn (Crown et al., 2002a). Stimulation of the tibialis anterior muscle still elicited a flexion response, but response-contingent shock did not produce an increase in flexion duration. Instead, subjects responded in a mechanical manner, with shock eliciting a robotic like response that often varied little over time. The consistency of responding was, in some cases, remarkable [e.g., varying less than 10% across consecutive training bins midway (min 16–20) through testing], seemingly affected only by motor fatigue.

**Figure 6:**
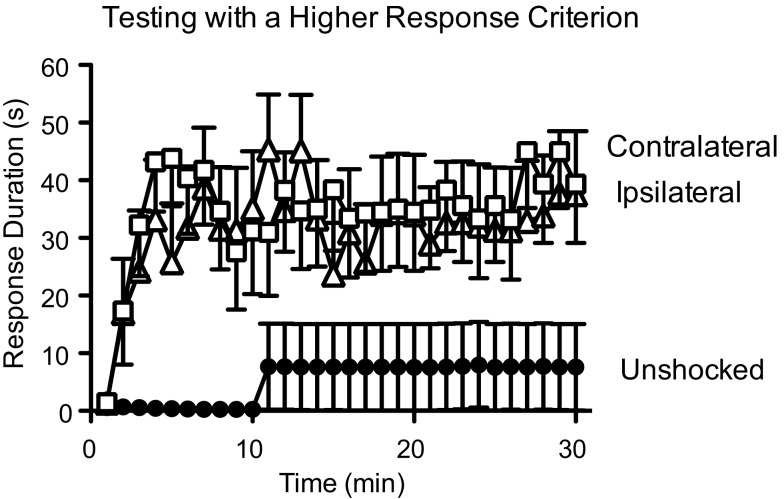
**Prior training with controllable shock enables learning**. Spinally transected rats received instrumental training using a moderate (4 mm contact electrode depth) and were then tested with response-contingent shock applied to the pretrained (ipsilateral) or opposite (contralateral) leg. Prior to testing, the task was made more difficult by raising the response criterion to an electrode depth of 8 mm. Under these conditions, previously untrained rats (Unshocked) failed to learn. Rats that had received instrumental training were able to learn and this was true irrespective of whether they were tested on the ipsilateral or contralateral leg. Adapted from Crown et al. (2002a).

An interesting feature of the learning deficit is that uncontrollable stimulation applied to one leg impairs learning when subjects are tested on the contralateral limb, an observation that suggests that uncontrollable stimulation induces a general change within the lumbosacral spinal cord that undermines (disables) the capacity for instrumental learning. Given this observation, Crown et al. (2002a) looked at whether controllable stimulation might have the opposite effect and act to enable learning. To examine this issue, subjects received 30 min of training using our usual response criterion, which submerged the contact electrode by 4 mm. We then tested subjects on either the same or opposite leg with a higher (8 mm) response criterion. Raising the criterion made the task so difficult that untrained subjects failed to learn (Figure 6). But subjects that had previously been trained with controllable shock learned at this high criterion and this was true independent of whether they were tested on the trained (ipsilateral) or untrained (contralateral) leg. It appears that training with controllable stimulation has an enabling effect that generally promotes instrumental learning.

Given that controllable and uncontrollable stimulation appear to impact spinal cord plasticity in an opposing manner, Crown and Grau (2001) explored whether the two forms of stimulation interact. Would, for example, prior training with controllable stimulation have a protective effect that prevents the induction of the learning deficit? To test this (see Table 1Ai), spinally transected rats received 30 min of training with controllable shock (Master), uncontrollable shock (Yoked), or nothing (Unshocked). Subjects then received 6 min of variable intermittent tail-shock, which we had previously shown produces a learning deficit (Crown et al., 2002b). Finally, subjects were tested with response-contingent shock applied to the untrained leg. As usual, subjects that had received intermittent tail-shock alone failed to learn. This learning deficit was not observed in rats that received controllable stimulation prior to non-contingent tail-shock, suggesting that training with controllable shock blocked the induction of the learning deficit.

**Table 1:**
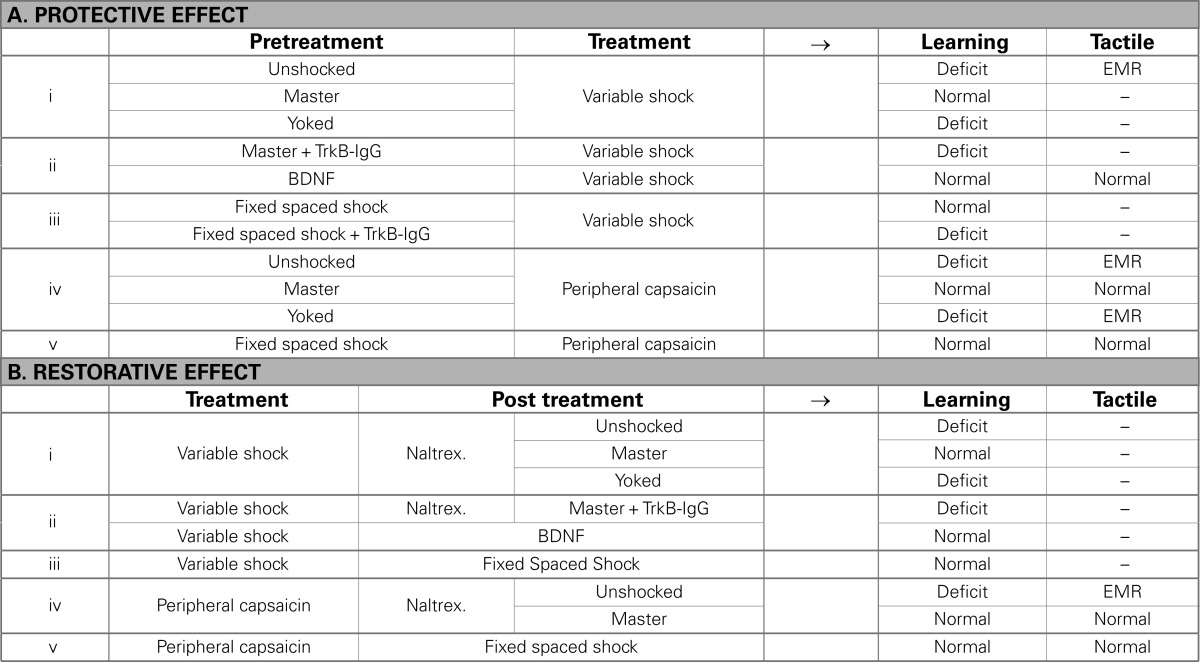
**Impact of instrumental training (i, ii, and iv), or an extended exposure to fixed spaced shock (iii and v), on the learning deficit and the enhanced mechanical reactivity (EMR) induced by variable shock (i and iii) or capsaicin (iv and v) treatment**.

Behavioral treatments have both a protective (A) and restorative (B) effect that reduces the learning deficit and EMR. Both the protective and the restorative effect of instrumental training have been linked to the release of BDNF (ii). To assess the restorative effect of instrumental training (B: i, ii, iv), naltrexone (Naltrex.) was given prior to training to block the expression of the learning deficit. Untested cells are indicated with a “–.”

Crown and Grau (2001) also explored whether training with controllable stimulation could have a restorative effect that reinstates the capacity for learning after the deficit has been induced. But how can we test this if uncontrollable stimulation disrupts subsequent learning? To explore the therapeutic potential of controllable stimulation, we needed a way of temporarily blocking the expression of the learning deficit. Concurrent studies had revealed that i.t. administration of an opioid antagonist (naltrexone) blocked the expression of the learning deficit (Joynes and Grau, 2004). Perhaps, if we trained rats while the deficit was pharmacologically blocked, behavioral training would have a long-term restorative effect that would be evident 24 h later (after the drug had cleared the system). The experimental design (Table 1Bi) was roughly the mirror image of the one used to examine the protective effect of controllable stimulation (Table 1Ai). First, we induced a learning deficit by exposing rats to variable intermittent tail-shock. Subjects then received an i.t. injection of naltrexone, followed by 30 min of training with controllable shock (Master), uncontrollable shock (Yoked), or nothing (Unshocked). The next day, rats were tested with controllable shock applied to the untrained leg. Rats that received uncontrollable shock alone failed to learn, confirming that non-contingent shock induces a lasting deficit and that the drug treatment *per se* had no long-term beneficial effect on performance. Importantly, rats that received non-contingent shock were able to learn when controllable stimulation was applied immediately after naltrexone treatment, confirming that the drug blocks the expression of the learning deficit. The critical question was whether this training would have a lasting therapeutic effect that would be evident the next day when subjects were tested in a drug-free state. We found that it did, suggesting that training with controllable stimulation can reverse the learning deficit.

**Figure 7:**
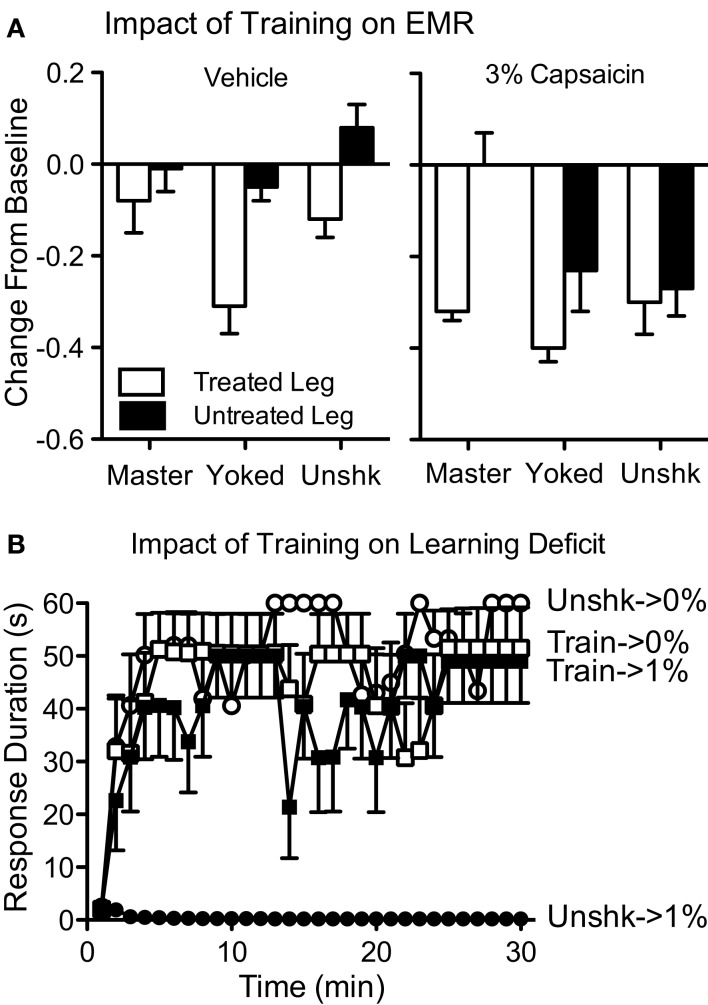
**Training with controllable shock attenuates the EMR and learning deficit observed after peripheral capsaicin**. **(A)** Spinally transected rats received controllable shock (Master), uncontrollable shock (Yoked) or nothing (Unshk). They then received an intradermal injection of capsaicin (3%) or its vehicle into the paw of the pretreated leg. Mechanical reactivity was tested using von Frey stimuli applied to the treated, or untreated, leg. In vehicle treated rats, uncontrollable shock enhanced reactivity on the treated leg. Capsaicin induced a robust bilateral EMR in both the Yoked and Unshk groups. Training with controllable stimulation (Master) eliminated the EMR observed on the contralateral (Untreated) leg. **(B)** Transected rats received instrumental training (Train) or nothing (Unshk), followed by a peripheral injection of capsaicin (1%) into the paw of the same leg. The next day, rats were tested with response-contingent shock applied to the contralateral leg. Untrained rats that received capsaicin (Unshk → 1%) failed to learn. Prior training with controllable shock (Train → 1%) eliminated this learning deficit. Adapted from Hook et al. (2008).

Earlier, we described how manipulations that induce central sensitization also impair instrumental learning. For example, intradermal capsaicin (a TRPV1 receptor agonist) produces both EMR and a lasting impairment of instrumental learning that is observed when subjects are tested 24 h later on the untreated (contralateral) leg (Hook et al., 2008). If capsaicin treatment and uncontrollable stimulation impact spinal plasticity in the same way, training with controllable stimulation should attenuate the capsaicin-induced learning deficit. To examine this, spinally transected rats received 30 min of training with controllable leg-shock (Master), uncontrollable leg-shock (Yoked), or nothing (Unshocked). Immediately after, they received an intradermal injection of 3% capsaicin or its vehicle to the same leg. Hook et al. (2008) then assessed mechanical reactivity (Figure 7A). In vehicle treated rats, uncontrollable, but not controllable, shock-induced EMR on the treated leg. Capsaicin produced a robust EMR in both the Unshocked and Yoked groups on both the treated and untreated leg. As noted by Ferguson et al. (under review), inflammatory agents can induce peripheral effects that contribute to the EMR observed on the ipsilateral leg. For this reason, the EMR observed on the contralateral (untreated) leg is often viewed as a purer measure inflammation-induced central sensitization. Given this, it is informative that prior training with controllable shock eliminated the EMR observed when subjects were tested on the untreated leg, but had no effect on reactivity when subjects were tested on the treated leg. A similar pattern was observed when peripheral inflammation was induced with intradermal formalin (Ferguson et al., under review). The next day, instrumental learning was tested using the untreated limb. Hook et al. (2008) found that a high concentration of capsaicin induced a robust learning deficit. Prior training with controllable stimulation appeared to lessen this deficit, but the effect was not robust. We reasoned that a small effect may have been observed because capsaicin produced such a strong learning impairment (Figure 5). To evaluate this possibility, we repeated the experiment using a lower concentration of capsaicin (1%). Subjects that received capsaicin alone (Unshk → 1%) failed to learn when tested on the contralateral leg 24 h later (Figure 7B). More importantly, prior training with controllable shock (Train → 1%) completely blocked the deficit.

Hook et al. (2008) then examined the converse issue, whether training with controllable stimulation could restore the capacity for learning if given *after* capsaicin treatment. Again, we faced a dilemma, because our behavioral rehabilitation depends on the capacity for learning, yet that was disrupted by capsaicin treatment. If uncontrollable shock and inflammation impair learning through a common mechanism, we should be able to block the expression of the learning deficit by pretreating subjects with naltrexone. To test this, rats received an intradermal injection of 1% capsaicin or its vehicle. Six hours later, half the subjects in each condition received an i.t. injection of naltrexone, followed by 30 min of training with response-contingent shock applied to the treated leg. We found that capsaicin induced a learning deficit and that the expression of this deficit was blocked by naltrexone. Hook et al. (2008) then tested the subjects 24 h later with response-contingent shock applied to the contralateral leg. We found that training with controllable stimulation had a therapeutic effect that restored the capacity for learning in capsaicin-treated rats (Table 1Biv).

Our results demonstrate that training with controllable stimulation induces a spinally mediated alteration that enables instrumental learning and exerts a protective effect that counters the learning deficit induced by either uncontrollable stimulation or peripheral inflammation.

### Neural mechanisms

We next consider the neural mechanisms that underlie instrumental learning and its protective/restorative effect. The first key question is: where does the learning occur? Liu et al. (2005) addressed this issue using a combination of techniques. We began by microinjecting fluorescent tracers (DiI and Fluoro-Gold) into the tibialis anterior muscle, at the site and depth of the needle electrode used to induce a flexion response. We found that the dyes labeled motoneurons in the lower L4-L5 region, an area implicated in the production of hind-limb stepping behavior (Nishimaru and Kudo, 2000). Next, separate groups of T2 transected rats received a slow infusion of lidocaine through an i.t. cannula positioned at T10/11, L3/4, S2, or Co1. Using India ink, we showed that this injection procedure impacted a region that extended approximately 0.1–0.2 cm rostral and 0.8–0.9 cm caudal to the cannula tip. When lidocaine was slowly infused, it disrupted performance when infused at L3-L4, but not at T10/11 or Co1 (with a partial effect when given at S2).

Liu et al. (2005) then examined the impact of selective knife cuts that transected the cord at different levels between L1 and S1, reasoning that spinal learning should remain intact as long as the knife cut is rostral to the essential circuit, while a transection at the site of learning would have a disruptive effect. We found that knife cuts between L1 and L4 had little effect on instrumental learning and that a more caudal cut, at L6-S1, disrupted learning. Finally, we combined a transection at L4 with a second more caudal transection, at S2, S3, or Co1. Learning was observed when the second transection occurred at S3 or lower, but not at S2, implying that the essential neural circuit lies between L4 and S3. These experiments both localize the essential neural circuit and laid the groundwork for future studies designed to identify the underlying neurochemical systems.

Given that NMDA-mediated plasticity has been shown to play an important role in a variety of learning phenomena (e.g., Morris et al., 1986; Collingridge and Bliss, 1987; Morris, 1994), and the discovery that spinal neurons support NMDAR-mediated plasticity (Dickenson and Sullivan, 1987; Coderre et al., 1993), we examined whether spinally mediated instrumental learning depends on the NMDAR. Using the competitive NMDAR antagonist AP5, Joynes et al. (2004) showed that learning was disrupted in a dose-dependent manner. Ferguson et al. (2006) subsequently extended this observation, demonstrating that learning is also disrupted by pretreatment with the non-competitive antagonist MK-801.

Further work showed that AP5 not only disrupts the acquisition of spinal learning, it also undermines the maintenance of instrumental behavior (Joynes et al., 2004). A similar outcome was reported for another example of NMDAR-dependent plasticity, wind-up (the enhancement in neural excitability observed with repetitive electrophysiological stimulation at an intensity that engages C-fibers; Mendell, 1966; Ma and Woolf, 1995). In this way, spinally mediated forms of NMDAR-mediated plasticity appear to differ from traditional preparations, such as hippocampal LTP, where it is generally held that NMDAR-dependent plasticity contributes to the induction, but not the maintenance, of LTP (Staubli et al., 1989).

**Figure 8:**
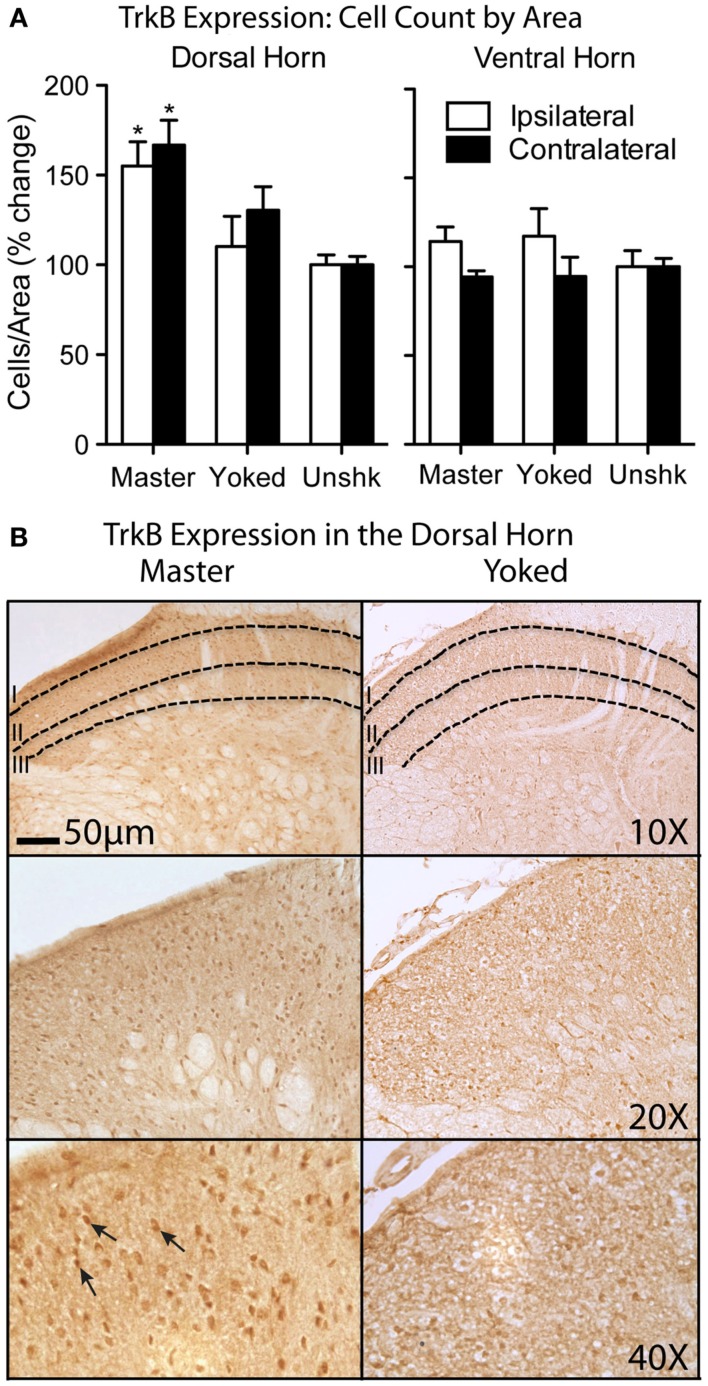
**Controllable shock increases TrkB expression within the dorsal horn**. **(A)** Number of cells labeled within the dorsal and ventral horn. Master rats exhibited greater TrkB expression within dorsal horn on both the treated (shocked; ipsilateral) and untreated side (contralateral). No differences were observed within the ventral horn (*p < 0.05). **(B)** Light immunohistochemistry showed increased TrkB immunolabeling in the dorsal horn of Master, but not Yoked, rats. Adapted from Huie et al. (2012b).

In collaboration with Fernando Gómez-Pinilla and Reggie Edgerton (Gómez-Pinilla et al., 2007), we conducted cellular assays that targeted genes implicated in plasticity. This study was motivated, in part, by the hypothesis that controllable stimulation might enable learning by engaging processes related to the release of BDNF. Research suggested that BDNF is essential to the development of LTP (Kang and Schuman, 1995; Patterson et al., 1996; Bekinschtein et al., 2008) and that this neurotrophin potentiates plasticity in spinal neurons (Heppenstall and Lewin, 2001; Baker-Herman et al., 2004; Zhou et al., 2008). To examine whether instrumental training affects the expression of BDNF, spinally transected rats were given controllable shock (Master), an equal amount of uncontrollable stimulation (Yoked), or nothing (Unshocked). After training, the L4-S1 spinal cord was removed and real-time RT-PCR was performed. We found that training with controllable stimulation produced a significant increase in BDNF mRNA expression, while uncontrollable stimulation produced a decrease (relative to the unshocked controls). Two down-stream targets, calcium/calmodulin activated protein kinase II (CaMKII) and the gene transcription factor cAMP-response element binding protein (CREB) showed the same pattern of results. We then examined whether instrumental performance predicted mRNA expression. Reasoning that expression may be most related to performance during the learning phase, we computed the mean response duration observed during the first 10 min of training. Independent analyses revealed that BDNF, CaMKII, and CREB were well-correlated with instrumental performance in master rats (all *r*’s > 0.93, *p* < 0.005). No significant relations were observed in the yoked controls. *In situ* hybridization showed that training with controllable shock enhanced BDNF mRNA expression throughout the spinal central gray (Huie et al., 2012b). Protein assays (Western blotting), showed that training with controllable shock increases the expression of both BDNF and its receptor, the trypomyosin receptor kinase TrkB. Immunohistochemical analyses revealed that controllable stimulation enhances TrkB protein expression within neurons of the dorsal horn (Figure 8), a modification that may provide a form of synaptic tag (Lu et al., 2011).

Given that the production of new protein will require some time, we hypothesized that the increase in BDNF and CaMKII expression may mediate the consequences of training, rather than instrumental learning *per se*. Supporting this, pretreatment with either a BDNF inhibitor (TrkB-IgG) or a CaMKII inhibitor (AIP) did not have a significant impact on instrumental learning (Gómez-Pinilla et al., 2007). After training, we tested subjects on the contralateral leg with a higher response criterion. As described above, in the absence of pretraining, subjects could not learn. Pretrained rats were able to learn when tested with a higher response criterion and this effect was blocked by pretreatment with either TrkB-IgG or AIP. Further evidence that BDNF contributes to the enabling effect was derived by administering BDNF (i.t.) prior to testing with a high response criterion. As a positive control, Gómez-Pinilla et al. (2007) also included a group that received instrumental training instead of drug treatment. As usual, these subjects were able to learn when tested with a higher criterion. Pretreatment with BDNF (0.1–0.4 μg i.t.) also enabled learning and did so in a dose-dependent manner.

**Figure 9:**
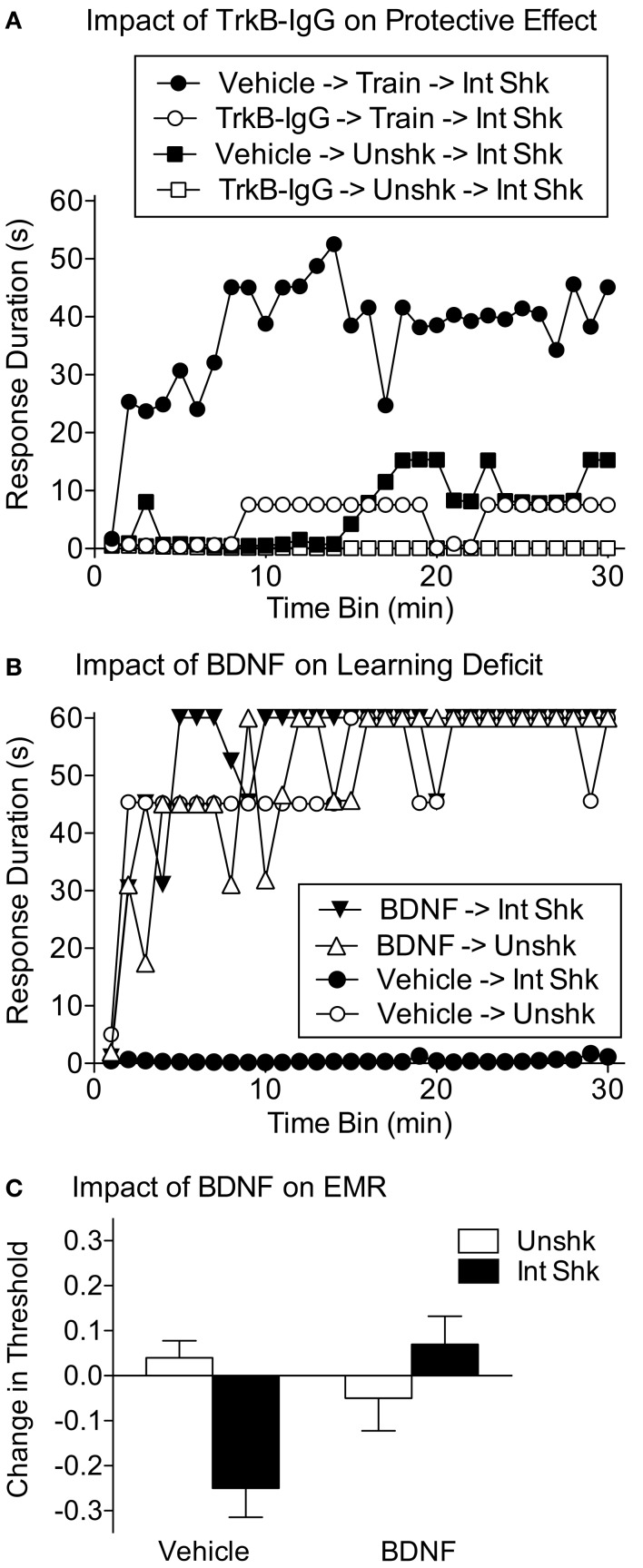
**Evidence BDNF contributes to the protective effect of controllable stimulation**. **(A)** Spinally transected rats received the BDNF inhibitor TrkB-IgG (i.t.) or its vehicle, followed by instrumental training (Train) or nothing (Unshk). Subjects then received variable intermittent tail-shock (Int Shk). The next day, subjects were tested with response-contingent shock applied to the previously untrained leg. Intermittent shock induced a learning deficit in the untreated subjects (Vehicle → Unshk → Int Shk). Prior training with controllable shock (Vehicle → Train → Int Shk) prevented the learning deficit. Pretreatment with TrkB-IgG (TrkB-IgG → Train → Shk) eliminated this protective effect. **(B)** Rats received BDNF (0.4 μg, i.t.) or its vehicle and 30 min later variable intermittent tail-shock (Int Shk) or nothing. Subjects were tested 24 h later. Vehicle treated rats that had received intermittent shock (Vehicle → Int Shk) failed to learn. Pretreatment with BDNF (BDNF → Int Shk) blocked the induction of this learning deficit. **(C)** Spinally transected rats received BDNF (0.4 μg, i.t.) or its vehicle followed by 6 min of variable intermittent shock (Int Shock) to one hind leg or nothing (Unshk). Mechanical reactivity was then tested using von Frey stimuli. Because comparable results were observed on both the shocked and unshocked leg, the data were collapsed across this variable. Intermittent shock-induced EMR in vehicle treated rats, but not rats pretreated with BDNF. Adapted from Huie et al. (2012b).

Huie et al. (2012b) then examined whether BDNF contributes to the protective/restorative effect of instrumental training. To evaluate whether BDNF was necessary to the protective effect, subjects were given TrkB-IgG or its vehicle. Subjects then received instrumental training, or nothing (Unshk), prior to 6 min of intermittent tail-shock (Int Shk). The next day, subjects were tested on the contralateral leg using the usual response criterion. In vehicle treated rats, prior training with controllable stimulation blocked the induction of the learning deficit. Pretreatment with TrkB-IgG eliminated this protective effect (Figure 9A).

If instrumental learning has a protective effect because it induces the release of BDNF, then i.t. BDNF should substitute for instrumental training and inhibit the induction of the learning deficit. To explore this possibility, Huie et al. (2012b) administered a low dose of BDNF (0.4 μg i.t.) or its vehicle 30 min before they received 6 min of intermittent tail-shock (Int Shk). The next day subjects were tested with response-contingent shock. As usual, uncontrollable shock impaired learning. No learning impairment was observed in rats that received BDNF prior to uncontrollable shock (Figure 9B).

Above we noted that exposure to uncontrollable stimulation enhances tactile reactivity. Huie et al. (2012b) replicated this finding and showed that pretreatment with BDNF also attenuates shock-induced EMR (Figure 9C). This observation contrasts with other studies that implicate BDNF in the induction of central sensitization (Kerr et al., 1999; Garraway et al., 2003; Merighi et al., 2008; Lu et al., 2009), a finding that suggests that BDNF should have, if anything, enhanced EMR. This apparent discrepancy is not an isolated instance. For example, Pezet et al. (2002) showed that treatment with BDNF can induce a thermal antinociception, an effect they attributed to BDNF inhibiting (via a GABAergic interneuron) substance P release within the dorsal horn. In a model of neuropathic pain (spinal nerve ligation), Lever et al. (2003) showed that i.t. BDNF attenuated the ligation-induced thermal hyperalgesia. Nerve injury was associated with a reduction in GABA, which was restored by BDNF treatment. In a similar vein, Cejas et al. (2000) showed that application of BDNF secreting cells a week after sciatic nerve injury attenuated both the injury-induced mechanical allodynia and thermal hyperalgesia. Importantly, both effects were observed for weeks after treatment. As noted in a recent review (Merighi et al., 2008), there is also ample evidence that BDNF can enhance pain. For example, Coull et al. (2005) showed that a high dose of BDNF (20 μg) can induce tactile allodynia. Conversely, Kerr et al. (1999) reported that treatment with TrkB-IgG attenuates the nociceptive responses elicited by intraplantar treatment with formalin or carrageenan. Similarly, mice that are BDNF deficient in nociceptive neurons exhibit diminished formalin-induced pain behavior (second phase) and attenuated thermal hyperalgesia after carrageenan (Zhao et al., 2006). These latter studies suggest that the induction of pain behavior after peripheral inflammation depends on endogenous BDNF.

Brain-derived neurotrophic factor likely yields a wide range of effects because it can influence neural processing within the spinal cord in multiple ways. First, it can act postsynaptically to enhance neural excitability through a NMDAR-mediated process. This action has been observed within nociceptive neurons in lamina II (Garraway et al., 2003) and in motoneurons of the ventral horn (Arvanian and Mendell, 2001). When coupled with response-contingent stimulation, we assume that this type of mechanism contributes to the BDNF-dependent enabling of instrumental learning (Gómez-Pinilla et al., 2007). Second, BDNF can act presynaptically to inhibit transmitter release, and this effect too has been observed within both the dorsal (Pezet et al., 2002) and ventral (Arvanian and Mendell, 2001) spinal cord. As noted above, this inhibitory effect has been attributed to a BDNF-dependent activation of GABAergic interneurons (Pezet et al., 2002). Given these observations, we suggest that the outcome observed depends upon at least three factors: (1) the dose of BDNF used [low concentrations appear to have an antinociceptive effect (Miki et al., 2000; Huie et al., 2012b) while a high concentration can enhance pain behavior (Miki et al., 2000; Coull et al., 2005)]; (2) the model of pain behavior employed; and (3) whether subjects have received a spinal injury (Garraway and Mendell, 2007). The third variable may be especially important because injury releases spinal mechanisms from sources of tonic inhibition (e.g., 5-HT), can alter levels of GABA, and increase the intracellular levels of Cl^−^ which can cause GABA to have a depolarizing effect (Millan, 2002; Diaz-Ruiz et al., 2007; Gwak and Hulsebosch, 2011). In this compromised state, we posit that BDNF may generally benefit spinal function, to curb over-excitation and promote adaptive plasticity.

A final complexity stems for the realization that the precursor (proBDNF) to the mature form of BDNF (mBDNF) is biologically active and can induce cellular effects that are antagonist to the action of mBDNF (Bothwell, 1996; Lee et al., 2001; Lu et al., 2005; Cunha et al., 2010), leading others to suggest a yin-yang model of proBDNF-mBDNF function (Lu et al., 2005). Though speculative, it is possible that the opposing effects of controllable and uncontrollable are related to the relative balance of proBDNF to mBDNF. At the least, some caution is warranted in clinical applications, because a physiological manipulation designed to increase BDNF protein expression could inadvertently lead to a maladaptive outcome if the conversion of proBDNF to mBDNF is down-regulated.

Huie et al. (2012b) also asked whether BDNF release plays an essential role in the therapeutic effect of controllable shock. Subjects were given 6 min of non-contingent tail-shock. To temporarily block the expression of the learning deficit, all subjects then received an i.t. injection of naltrexone. To examine whether the therapeutic effect of training depends on BDNF, half the subjects also received the BDNF inhibitor TrkB-IgG. Finally, half the subjects in each drug condition received 30 min of training with response-contingent shock. The next day, subjects were tested for 30 min with response-contingent shock applied to the contralateral leg. As usual, uncontrollable shock impaired learning. Subjects that received 30 min of instrumental training after uncontrollable shock did not exhibit a learning deficit and this therapeutic effect of training was blocked by pretreatment with TrkB-IgG (Table 1Bii). Interestingly, a follow-up experiment showed that administering TrkB-IgG after instrumental training also blocked the therapeutic effect of training, suggesting that higher levels of BDNF must be maintained after training for it to have a lasting effect.

If training has a therapeutic effect because it increases BDNF release, then administration of BDNF should substitute for instrumental training and restore the capacity for learning. Huie et al. (2012b) examined this issue in two ways. In both experiments, subjects received uncontrollable tail-shock and were tested with response-contingent shock 24 h later. In the first experiment, BDNF was administered immediately after subjects received uncontrollable shock. In the second experiment, BDNF was given the next day, 30 min before testing. In both cases, BDNF treatment eliminated the learning deficit, suggesting that this neurotrophin can both reverse, and restore, the capacity for learning.

In summary, we have shown that instrumental learning depends on neurons that lie within the L4-S2 spinal tissue. Learning depends on a form NMDAR-mediated plasticity and engages the expression of a number of plasticity related genes, including BDNF, CaMKII, and CREB. We further showed that training with controllable stimulation increases the expression of both BDNF and its receptor, TrkB. The latter effect was localized to the dorsal horn. Finally, evidence was presented that the beneficial/restorative effect of instrumental training is related to the release of BDNF; a BDNF inhibitor (TrkB-IgG) blocked the protective/therapeutic effect of instrumental training and i.t. administration of BDNF substituted for instrumental training to both prevent, and reverse, the learning deficit.

## Predictability

Our focus has been on behavioral control and how it can engage an adaptive, BDNF-dependent, process that exerts a protective/restorative effect. Recently, Baumbauer et al. (2008, 2009a) discovered that temporal predictability can have a similar effect and may do so using some of the same neurobiological mechanisms. The original aim of these experiments was to identify the stimulus conditions that produce a learning deficit. Using electrophysiological stimulation of the sciatic nerve, Baumbauer et al. (2008) showed that 180 shocks at 0.5 Hz (an ISI of 2″) produces a deficit when shock intensity is increased to a level that engages C-fibers. Moreover, stimulation induced a deficit independent of whether it occurred in a variable (0.2–3.8 s apart, rectangular distribution) or regular (fixed spaced) manner. What was surprising is that, when shock number was increased fivefold (900 shocks), only variable shock impaired subsequent learning. Because we had previously shown that 180 fixed spaced shocks induce a deficit, the fact 900 fixed spaced shocks does not implies that the additional (720) stimuli engaged a restorative process that eliminated the learning deficit.

In a subsequent paper, Baumbauer et al. (2009a) showed that an extended exposure [24–30 min (720+ shocks)] to fixed spaced shock has a protective/restorative effect that parallels the beneficial effect of instrumental control (see Tables 1iii,v). Specifically, we found that 720 fixed spaced shocks given before, or after, 180 variably spaced shocks eliminates the learning deficit. Likewise, the learning deficit and EMR induced by peripheral capsaicin was attenuated by exposure to fixed spaced shock (Baumbauer et al., 2010; Baumbauer and Grau, 2011).

Other studies showed that an extended exposure to fixed spaced shock has a lasting protective effect that prevents the induction of the learning deficit by variably spaced shock given 24 h later (Baumbauer et al., 2009a). Rats given the NMDA antagonist MK-801 prior to fixed spaced stimulation do not exhibit the protective effect 24 h later, when challenged with variably spaced shock. The long-term protective effect is also eliminated by administering the protein synthesis inhibitor cycloheximide immediately after exposure to fixed spaced shock. Like behavioral control, pretreatment with the BDNF inhibitor TrkB-IgG eliminated the protective effect.

The observation that fixed and variably spaced shock have divergent effects on spinal function suggests that they are somehow discriminated; that introducing a regular (predictable) temporal relation engages distinct neural processes. Here, we need not take a stand on whether this discrimination involves a sensory filter or a central integrative process, possibly linked to the central pattern generator (CPG) assumed to organize stepping (Grillner and Wallen, 1985). What is important for present purposes is that the results imply that the consequences of intermittent stimulation depend on *both* controllability and predictability: controllable/predictable stimulation engages a BDNF-dependent process that appears to have a protective/restorative effect whereas uncontrollable/unpredictable stimulation engages processes that inhibit learning and enhance mechanical reactivity.

The fact that the long-term consequences of fixed spaced stimulation require extended training, are NMDA-dependent, and involve protein synthesis, suggests that a kind of learning may be engaged. In intact animals, there is ample evidence that elapsed time can act as a Pavlovian CS and, with a regularly presented US, elicit a CR that is timed to the occurrence of the US (temporal conditioning). This type of learning may underlie the fixed spaced shock effects described by Baumbauer et al. (2008, 2009a) and Baumbauer and Grau (2011).

In terms of clinical application, fixed spaced stimulation may provide an attractive alternative in situations where instituting behavioral control is not possible. There is a caveat, however, because far more training is needed to establish the fixed spaced shock effect; whereas the behavioral effects of controllable stimulation are evident within minutes of training (with fewer than 180 shocks), the beneficial effect of fixed spaced shock only emerges after extended training (e.g., 720 stimulus presentations or more). The spinal learning system appears to be better equipped (biologically prepared) to learn about behavioral controllability (i.e., contingent vs. non-contingent stimulation) than to learn about temporal predictability (i.e., fixed-space stimulation).

## Clinical Implications

**Figure 10:**
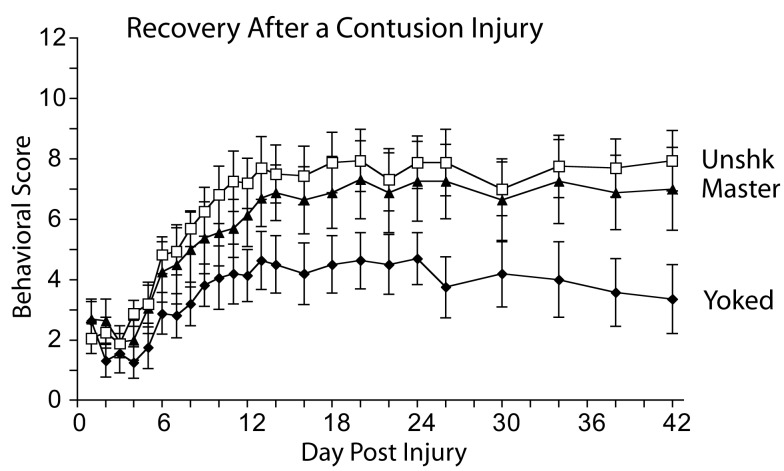
**Only uncontrollable stimulation impairs recovery after a contusion injury**. Rats received a moderate contusion injury and, 24 h later, 30 min of training with controllable shock (Master). Other groups received shock independent of leg position (Yoked) or nothing (Unshk). These treatments were repeated the next day and locomotor recovery was monitored for the next 6 weeks using a modified version of the BBB locomotor scale (Basso et al., 1995; Ferguson et al., 2004). Exposure to uncontrollable shock (Yoked) impaired recovery. Nociceptive stimulation had no adverse effect when it was given in a controllable manner (Master). Adapted from Grau et al. (2004).

We have begun to explore some of the clinical implications of our work and have shown that the same shock schedule (6 min of intermittent tail-shock) that impairs spinal plasticity also disrupts recovery after a contusion injury (Grau et al., 2004). A key question is whether this effect is also modulated by instrumental control. To explore this possibility, we administered a moderate contusion injury in the lower thoracic region. The next day, master rats received 30 min of response-contingent leg-shock (Master), while yoked subjects received an equal amount of shock given independent of leg position. A third group served as the unshocked controls. These treatments were repeated the next day and locomotor recovery was monitored over the next 6 weeks. We found that uncontrollable stimulation impaired recovery (Figure 10). Master subjects, that received the same amount of shock but could control its presentation, exhibited normal recovery. Thus, introducing instrumental control can blunt the adverse effect of nociceptive stimulation.

Other recent data suggest that the adverse effect of uncontrollable stimulation may be related to a down-regulation of BDNF. In these studies, rats again received a moderate contusion injury and uncontrollable shock 24 h later (Garraway et al., 2011). A day after shock treatment, subjects exhibited a decrease in BDNF mRNA and protein expression within the dorsal horn. Shock also down-regulated TrkB and CaMKII protein within the dorsal, but not the ventral, cord.

These observations suggest that our work using spinally transected rats has implications for recovery after a contusion injury. Our hope is to show that introducing instrumental control not only counters the effect of nociceptive stimulation, but also engages a BDNF-dependent process that promotes recovery. We suspect that demonstrating such an effect will require training parameters that minimize the unconditioned (unlearned) adverse effects of nociceptive stimulation. Accomplishing this may require a procedure in which shock intensity is titrated downward to the lowest level that supports learning.

Learning-like adaptations also impact stepping after injury (Edgerton et al., 2004). A particularly good example of this was reported by Edgerton et al. (1997), who showed that spinally transected animals can exhibit a training-induced alteration in hind-limb stepping. After subjects were trained to step on a treadmill, an obstacle was introduced – a bar that one paw struck during the swing phase. Over time, subjects exhibited a stronger flexion response during the swing phase, which reduced the force with which the paw hit the bar. Here too, the onset of a biologically significant stimulus (hitting the bar), in the presence of cues that signal a particular leg position, engenders a change in on-going behavior. Conversely, stand training appears to induce an effect that inhibits learning; rats that received 7 weeks of stand training exhibit impaired learning on a spinally mediated instrumental learning task (Bigbee et al., 2007). Our work also fits nicely with studies demonstrating that up-regulating BDNF expression can promote locomotor behavior in spinally transected rats (Boyce et al., 2007, 2012).

As discussed in Hook and Grau (2007), learning can also contribute to the behavioral changes elicited by the functional electrical stimulation (FES) used to prevent foot drop and/or muscle atrophy. Importantly, the stimulation used in FES is generally applied in a response-contingent manner (e.g., to drive cycling). Our work suggests that, if it was not, the stimulation could adversely affect spinal function.

Finally, Harkema et al. (2011) found that coupling epidural stimulation of the L5-S1 region with sensory stimulation related to bilateral extension and loading fostered standing behavior in a paraplegic patient (Harkema et al., 2011). In addition, when combined with task-specific sensory cues, epidural stimulation generated locomotor-like behavior. The researchers hypothesized that the stimulation was effective because it engaged populations of interneurons that integrate load-bearing related proprioceptive input to coordinate motor pool activity, and thereby enables use-dependent plasticity.

## A Summary Model

We have provided evidence that spinal neurons are sensitive to response-outcome relations and that this learning has a lasting effect, demonstrating that training with controllable stimulation enables learning whereas training with uncontrollable stimulation has a disabling effect that inhibits learning. We have further shown that these effects depend on NMDAR-dependent alterations within the spinal cord and that both effects have a general impact on plasticity. As discussed in Ferguson et al. (under review), because these effects concern factors that regulate the plasticity of plasticity, they can be considered forms of metaplasticity (Abraham and Bear, 1996).

**Figure 11:**
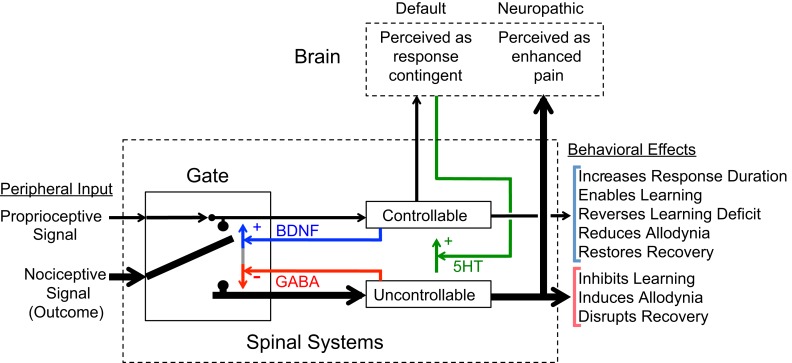
**A model of the processes that underlie the spinal consequences of controllable versus uncontrollable stimulation**. It is assumed that proprioceptive signals provide an indication of current limb position and that the system is biologically prepared to detect the relationship between this cue and the onset of a nociceptive stimulus (preparedness is represented by initial position of the nociceptive input, which is tilted in favor of behavioral control). When a relation is detected, the stimulation is encoded as controllable. This process promotes adaptive behavior (e.g., an increase in response duration), enables learning, prevents and reverses the learning deficit, attenuates the allodynia elicited by uncontrollable shock or peripheral inflammation, and prevents nociceptive stimulation from adversely affecting recovery. Findings reported above suggest that these adaptive processes are linked to BDNF (blue), which could enable learning and attenuate the consequences of uncontrollable stimulation by biasing the gate in favor of controllability. Uncontrollable stimulation appears to have the opposite effect, engaging a process (red) that inhibits learning, induces allodynia, and undermines recovery after a contusion injury. Psychologically, these maladaptive effects could lead to enhanced (neuropathic) pain. GABAergic systems have been shown to play an important role in both the induction and expression of the learning deficit (Ferguson et al., 2003). Recent data also implicate the cytokine TNF-alpha (Huie et al., 2012a). For both controllable and uncontrollable stimulation, NMDAR-mediated plasticity may provide a kind of physiological latch that maintains these states over time, yielding a form of metaplasticity that enables (controllable) or disables (uncontrollable) adaptive learning. In the uninjured state, descending serotonergic (5-HT) systems (green) counter the effects of uncontrollable stimulation, which we assume helps to maintain the default state (biased in favor of adaptive plasticity).

Our behavioral analysis sought to both clarify the nature of the learning and its relevance to rehabilitation and suggested that the key events are tied to the onset of nociceptive stimulation. Based on this observation, we suggested that the detection of control must be linked to proprioceptive signals and hypothesized that the system is biased in favor of control. This process can be envisioned as a kind of physiological gate, in which the relationship between the nociceptive stimulus and proprioceptive signals determines how stimulation affects spinal systems (Figure 11). If the nociceptive signal is tied to a particular proprioceptive signal (controllable), it engages an adaptive behavioral response (that reduces net exposure to the nociceptive signal) and enlists down-stream (BDNF-dependent) processes that exert a protective/restorative effect. If the stimulus occurs in a manner that is unrelated to a particular proprioceptive cue (uncontrollable), it engages an opponent-like process that inhibits new learning, induces EMR, and impairs recovery. The induction and expression of this deficit has been linked to a GABA-dependent process. Opioids and NMDAR/mGluR-mediated plasticity have also been shown to play a role. Finally, evidence suggests that in uninjured subjects descending 5-HT systems exert a protective effect that acts to counter the adverse effect of uncontrollable stimulation. Within this hypothetical system, NMDAR-mediated plasticity could contribute to long-term retention in a variety of ways. One possibility is that it acts as a kind of latch, locking the hypothetical gate in one mode or the other.

The model illustrated in Figure 11 was designed to illustrate the functional relations that underlie spinally mediated learning and how this affects nociceptive processing. The aim was to describe a system that could provide an interface between clinical application and the analysis of the underlying neurobiological mechanisms. While we believe that the switching metaphor provides a useful heuristic, it should be recognized that many details remain to be specified. Further, in seeking parsimony, we have likely consolidated functions that are mediated by distinct neuroanatomical systems. For example, we assume that abstracting the relation between proprioceptive and nociceptive inputs requires a form of neural convergence and reflects a local effect. Where might this convergence occur? Proprioceptive afferents that carry information regarding muscle length/velocity (A-alpha fibers) project to lamina VI, as well as deeper laminae (Watson et al., 2008). Nociceptive fibers (A-delta and C) project to laminae I and II. In addition, lamina V receives input from A-delta fibers and polysynaptic inputs from C-fibers. These anatomical considerations suggest that the abstraction of the response-outcome (proprioceptive-nociceptive) relation may occur in laminae V/VI. Alternatively, an interneuronal projection could relay proprioceptive signals to regions within the superficial dorsal horn that receive nociceptive input.

While we assume that learning the relation between a particular leg position and the onset of a nociceptive stimulus is mediated by a local interaction, our results suggest that some consequences of this learning have a more general effect that promotes learning and counters the adverse effects of uncontrollable stimulation. We have shown that this process depends on an up-regulation of BDNF and *in situ* hybridization suggests that BDNF mRNA expression is diffusely increased throughout both the dorsal and ventral horn (Huie et al., 2012b). Likewise, the consequences of uncontrollable stimulation have been likened to the induction of a diffuse state of over-excitation that generally saturates plasticity and enhances mechanical reactivity (Ferguson et al., 2006). This diffuse state has been tied to a GABA-dependent process and the cytokine TNF-alpha (Ferguson et al., 2003; Huie et al., 2012a). Thus, while we illustrate the consequences of these processes on a local effect (influencing the state of the hypothetical gate), we envision the metaplastic effects as having a more global influence on neural processing that extends across multiple laminae. Further, distinct components of the nociceptive signal may be important for learning and the induction of the learning deficit. Because learning depends on strong *R*-*O* contiguity, fast (myelinated) A-delta fiber input may be critical to abstracting the relation between proprioceptive and nociceptive inputs. At the same time, research indicates that C-fiber input is essential to the induction of the learning deficit (Baumbauer et al., 2008, 2009b).

Two additional details that need to be elucidated concern the mechanisms that underlie the dissemination of the metaplastic effects and the role of GABA. Our behavioral and cellular studies suggest that controllable/uncontrollable stimulation can affect neural processing of afferent signals from remote dermatomes. What process allows the functional spread of the cellular effect across distinct regions of the spinal cord? One possibility is that a cytokine (e.g., TNF-alpha) released from glia has a diffuse effect (Huie et al., 2012a; Vichaya et al., 2009). A second, and potentially related, question concerns the role of GABA. While it is clear that a GABA-dependent process can disrupt learning, it is not clear whether this is due to neural inhibition or a paradoxical excitatory effect linked to an injury-induced shift in intracellular chloride levels that causes GABA to have depolarizing effect (which could contribute to the saturation of NMDAR-mediated plasticity; Diaz-Ruiz et al., 2007; Gwak and Hulsebosch, 2011).

Our model is consistent with an emerging view of motor function. Postural control and adaptation to changing loads requires an internal model of limb dynamics that encodes proprioceptive information (Windhorst, 2007). Researchers have traditionally assumed that this model is mediated by supraspinal structures. However, data collected over the last 20 years has shown that spinal mechanisms can organize well-behaved dynamic limb movements in the absence of input from the brain. Given this, Windhorst (2007) has suggested that spinal systems must also build/maintain a motor map that is linked to proprioceptive/cutaneous input. It is further suggested that learning can occur within this system through a form of back-propagation in the dendritic tree of motoneurons, which could support NMDAR-dependent/Hebbian synaptic plasticity. From our view, the dynamic updating of an internal map could be seen as a form of instrumental learning.

It is also recognized that the model described above incorporates features of the gate control theory of pain (Melzack and Wall, 1965), which proposed that non-nociceptive input conducted by large myelinated fibers can inhibit pain. Our proposal extends this view by suggesting nociceptive inputs can also be modulated by proprioceptive cues. We further suggest that the consequences of non-nociceptive input will depend upon whether it is correlated with the onset of nociceptive stimulation. We also propose that the gate can be latched in one state or the other, providing a kind of sensory memory that will influence how subsequent nociceptive stimuli are processed.

Our research shows that procedures and constructs derived from the field of learning can help us understand how spinal neurons process neural signals. Our behavioral analyses uncovered the events that support learning and thereby shifted our view of how response-contingent stimulation is encoded, to see behavioral control as a form of sensor processing. Behavioral analyses further revealed how factors such as controllability and predictability can engage modulatory (metaplastic) effects that regulate adaptability and the development/maintenance of central sensitization. Our behavioral observations were reinforced by neurobiological studies that linked these effects to the neurotrophin BDNF and NMDAR-mediated plasticity. The studies suggest that behavioral factors can determine whether nociceptive signals lead to neuropathic pain and adversely affect recovery.

## Conflict of Interest Statement

Dr. Eric Crown receives salary and other financial incentives as an employe of Abbott Laboratories. The content of this review reflects Dr. Crown’s opinions and not those of Abbott Laboratories. No other author has a financial or commercial conflicts of interest to disclose.

## References

[B1] Abraham W. C. Bear M. F. (1996). Metaplasticity: the plasticity of synaptic plasticity. Trends Neurosci. 19, 126–130 10.1016/S0166-2236(96)80018-X 8658594

[B2] Arvanian V. L. Mendell L. M. (2001). Acute modulation of synaptic transmission to motoneurons by BDNF in the neonatal rat spinal cord. Eur. J. Neurosci. 14, 1800–1808 10.1046/j.0953-816x.2001.01811.x 11860475

[B3] Baker-Herman T. L. Fuller D. D. Bavis R. W. Zabka A. G. Golder F. J. Doperalski N. J. Johnson R. A. Watters J. J. Mitchell G. S. (2004). BDNF is necessary and sufficient for spinal respiratory plasticity following intermittent hypoxia. Nat. Neurosci. 7, 48–55 10.1038/nn1166 14699417

[B4] Basso D. M. Beattie M. S. Bresnahan J. C. (1995). A sensitive and reliable locomotive rating scale for open field testing in rats. J. Neurotrauma 12, 1–21 10.1089/neu.1995.12.1 7783230

[B5] Baumbauer K. M. Grau J. W. (2011). Timing in the absence of supraspinal input III: regularly spaced cutaneous stimulation prevents and reverses the spinal learning deficit produced by peripheral inflammation. Behav. Neurosci. 125, 37–45 10.1037/a0022009 21319886 PMC3074430

[B6] Baumbauer K. M. Hoy K. C. Huie J. R. Hughes A. J. Woller S. Puga D. A. Setlow B. Grau J. W. (2008). Timing in the absence of supraspinal input I: variable, but not fixed, spaced stimulation of the sciatic nerve undermines spinally-mediated instrumental learning. Neuroscience 155, 1030–1047 10.1016/j.neuroscience.2008.07.003 18674601 PMC2633135

[B7] Baumbauer K. M. Huie J. R. Hughes A. J. Grau J. W. (2009a). Timing in the absence of supraspinal input II: regular spaced stimulation induces a lasting alteration in spinal function that depends on the NMDA receptor, BDNF release, and protein synthesis. J. Neurosci. 29, 14383–14393 10.1523/JNEUROSCI.3583-09.2009 19923273 PMC2800823

[B8] Baumbauer K. M. Young E. E. Joynes R. L. (2009b). Pain and learning in a spinal system: contradictory outcomes from common origins. Brain Res Rev. 61, 124–143 10.1016/j.brainresrev.2009.05.004 19481111

[B9] Baumbauer K. M. Puga D. A. Lee K. H. Woller S. A. Hughes A. J. Grau J. W. (2010). Regularly Spaced Low Frequency Electrical Stimulation Dampens Tactile Reactivity and Attenuates Capsaicin-Induced Allodynia. 2010 Neuroscience Meeting Planner. San Diego, CA: Society for Neuroscience

[B10] Baumbauer K. M. Young E. E. Hoy K. C. Joynes R. L. (2006). Intrathecal infuions of anisomycin impact the learning deficit but not learning effect observed in spinal rats that received instrumental training. Brain Res. 173, 299–309 10.1016/j.bbr.2006.06.041 16914213

[B11] Beggs A. L. Steinmetz J. E. Patterson M. M. (1985). Classical conditioning of a flexor nerve response in spinal cats: effects of tibial nerve CS and a differential conditioning paradigm. Behav. Neurosci. 99, 496–508 10.1037/0735-7044.99.3.496 3843723

[B12] Bekinschtein P. Cammarota M. Izquierdo I. Medina H. (2008). BDNF and memory formation and storage. Neuroscientist 14, 147–156 10.1177/1073858407305850 17911219

[B13] Bigbee A. J. Crown E. D. Ferguson A. R. Roy R. R. Tillakaratne N. J. K. Tobin A. J. Grau J. W. Edgerton V. R. (2007). Two chronic motor training paradigms differentially alter the potential to perform a novel motor learning task in spinally transected rats. Behav. Brain Res. 180, 95–101 10.1016/j.bbr.2007.02.029 17434606 PMC2234650

[B14] Bothwell M. (1996). p75NTR: a receptor after all. Science 272, 506–507 10.1126/science.272.5261.506 8614797

[B15] Boyce V. S. Park J. Gage F. H. Mendell L. M. (2012). Differential effects of brain derived neurotrophic factor and neutrophin-3 on hindlimb function in paraplegic rats. Eur. J. Neurosci. 35, 221–232 10.1111/j.1460-9568.2011.07950.x 22211901 PMC3509221

[B16] Boyce V. S. Tumolo M. Fischer I. Murray M. Lemay M. A. (2007). Neurotrophic factors promote and enhance locomotor recovery in untrained spinalized cats. J. Neurophysiol. 98, 1988–1996 10.1152/jn.00391.2007 17652412

[B17] Buerger A. A. Chopin S. F. (1976). Instrumental avoidance conditioning in spinal vertebrates. Adv. Psychobiol. 3, 437–461 788482

[B18] Buerger A. A. Fennessy A. (1970). Long-term alteration of leg position due to shock avoidance by spinal rats. Nature 225, 751–752 10.1038/225751a0 5547250

[B19] Cejas P. J. Martinez M. Karmally S. McKillop M. McKillop J. Plunkett J. A. Oudega M. Eaton M. J. (2000). Lumbar transplant of neurons genetically modified to secrete brain-derived neurotrophic factor attenuates allodynia and hyperalgesia after sciatic nerve constriction. Pain 86, 195–210 10.1016/S0304-3959(00)00245-1 10779676

[B20] Chopin S. F. Buerger A. A. (1976). Instrumental avoidance conditioning in the spinal rat. Brain Res. Bull. 1, 177–183 10.1016/0361-9230(76)90067-8 974801

[B21] Church R. M. (1964). Systematic effect of random error in the yoked control design. Psychol. Bull. 62, 122–131 10.1037/h0042733 14199645

[B22] Church R. M. (1989). “The yoked control design,” in Aversion, Avoidance, and Anxiety: Perspectives on Aversively Motivated Behavior, eds Archer T. Nilsson L. (Hillsdale, NJ: Erlbaum), 403–415

[B23] Church R. M. Lerner N. D. (1976). Does the headless roach learn to avoid? Physiol. Psychol. 4, 439–442

[B24] Coderre T. J. Katz J. Vaccarino A. L. Melzack R. (1993). Contribution of central neuroplasticity to pathological pain: review of clinical and experimental evidence. Pain 52, 259–285 10.1016/0304-3959(93)90161-H 7681556

[B25] Collingridge G. L. Bliss T. V. P. (1987). NMDA receptors and their role in long-term potentiation. Trends Neurosci. 10, 288–293 10.1016/0166-2236(87)90175-5

[B26] Coull J. A. Beggs S. Boudreau D. Boivin D. Tsuda M. Inoue K. Gravel C. Salter M. W. De Koninck Y. (2005). BDNF from microglia causes the shift in neuronal anion gradient underlying neuropathic pain. Nature 438, 1017–1021 10.1038/nature04223 16355225

[B27] Crown E. D. Ferguson A. R. Joynes R. L. Grau J. W. (2002a). Instrumental learning within the spinal cord: II. Evidence for central mediation. Physiol. Behav. 77, 259–267 10.1016/S0031-9384(02)00859-4 12419402

[B28] Crown E. D. Joynes R. L. Ferguson A. R. Grau J. W. (2002b). Instrumental learning within the spinal cord: IV. Induction and retention of the behavioral deficit observed after noncontingent shock. Behav. Neurosci. 116, 1032–1051 10.1037/0735-7044.116.6.1032 12492302

[B29] Crown E. D. Grau J. W. (2001). Preserving and restoring behavioral potential within the spinal cord using an instrumental training paradigm. J. Neurophysiol. 86, 845–855 11495955 10.1152/jn.2001.86.2.845

[B30] Crown E. D. Grau J. W. (2005). Evidence that descending systems protect behavioral plasticity against the disruptive effect of nociceptive stimulation. Exp. Neurol. 196, 164–176 10.1016/j.expneurol.2005.07.016 16139268

[B31] Cunha C. Brambilla R. Thomas K. L. (2010). A simple role for BDNF in learning and memory. Front. Mol. Neurosci. 3:1 10.3389/neuro.02.001.2010 20162032 PMC2821174

[B32] Davies J. E. Marsden C. A. Roberts M. H. T. (1983). Hyperalgesia and reduction of monoamines resulting from lesions of the dorsolateral funiculus. Brain Res. 261, 59–68 10.1016/0006-8993(83)91283-0 6188514

[B33] Diaz-Ruiz A. Salgado-Ceballos H. Montes S. Maldonado V. Tristan L. Alcaraz-Zubeldia M. Ríos C. (2007). Acute alterations of glutamate, glutamine, GABA, and other amino acids after spinal cord contusion in rats. Neurochem. Res. 32, 57–63 10.1007/s11064-006-9225-5 17160506

[B34] Dickenson A. H. Sullivan A. F. (1987). Evidence for a role of the NMDA receptor in the frequency dependent potentiation of deep rat dorsal horn nociceptive neurons following C fibre stimulation. Neuropharmacology 26, 1235–1238 10.1016/0028-3908(87)90275-9 2821443

[B35] Domjan M. (2010). Principles of Learning and Behavior, 6th Edn. Belmont, CA: Wadsworth

[B36] Durkovic R. G. (1986). “The spinal cord: a simplified system for the study of neural mechanisms of mammalian learning and memory,” in Development and Plasticity of the Mammalian Spinal Cord, eds Goldberger M. E. Gorio A. Murray M. (Padova: Liviana Press), 2921–2925

[B37] Durkovic R. G. (2001). “Pavlovian conditioning of flexion reflex potentiation in spinal cat: temporal effects following spinal transection,” in Spinal Cord Plasticity: Alterations in Reflex Function, eds Patterson M. M. Grau J. W. (Norwell, MA: Kluwer Academic Publishers), 55–76

[B38] Eaton M. J. Santiago D. I. Dancausse H. A. Whittemore S. R. (1997). Lumbar transplants of immortalized serotonergic neurons alleviate chronic neuropathic pain. Pain 72, 59–69 10.1016/S0304-3959(97)00015-8 9272788

[B39] Edgerton V. R. Roy R. R. de Leon R. Tillakaratne N. Hodgson J. A. (1997). Does motor learning occur in the spinal cord? Neuroscientist 3, 287–294

[B40] Edgerton V. R. Tillakartne N. J. Bigbee A. J. de Leon R. D. Roy R. R. (2004). Plasticity of the spinal neural circuitry after injury. Annu. Rev. Neurosci. 27, 145–167 10.1146/annurev.neuro.27.070203.144308 15217329

[B41] Estes W. K. (1944). An experimental study of punishment. Psychol. Monogr. 57, No 3 (Whole No. 263). 10.1037/h0093550

[B42] Estes W. K. (1969). “Outline of a theory of punishment,” in Punishment and Aversive Behavior, eds Campbell B. A. Chruch R. M. (New York: Appleton-Century-Crofts), 57–82

[B43] Faden A. I. Gannon A. Basbaum A. I. (1988). Use of serotonin immunocytochemistry as a marker of injury severity after experimental spinal trauma in rats. Brain Res. 450, 94–100 10.1016/0006-8993(88)91548-X 3401725

[B44] Ferguson A. R. Bolding K. A. Huie J. R. Hook M. A. Santillano D. R. Miranda R. C. Grau J. W. (2008). Group I metabotropic glutamate receptors control metaplasticity of spinal cord learning through a PKC-dependent mechanism. J. Neurosci. 28, 11939–11949 10.1523/JNEUROSCI.3098-08.2008 19005059 PMC2628285

[B45] Ferguson A. R. Crown E. D. Grau J. W. (2006). Nociceptive plasticity inhibits adaptive learning in the spinal cord. Neuroscience 141, 421–431 10.1016/j.neuroscience.2006.03.029 16678969

[B46] Ferguson A. R. Hook M. A. Garcia G. Bresnahan J. C. Beattie M. S. Grau J. W. (2004). A simple transformation that improves the metric properties of the BBB scale. J. Neurotrauma 21, 1601–1613 10.1089/neu.2004.21.1601 15684652

[B47] Ferguson A. R. Washburn S. N. Crown E. D. Grau J. W. (2003). GABAA receptor activation is involved in non-contingent shock inhibition of instrumental conditioning in spinal rat. Behav. Neurosci. 117, 799–812 10.1037/0735-7044.117.2.263 12931964

[B48] Fitzgerald L. A. Thompson R. F. (1967). Classical conditioning of the hindlimb flexion reflex in the acute spinal cat. Psychon. Sci. 47, 345–351

[B49] Garcia J. Brett L. P. Rusiniak K. W. (1989). “Limits of Darwinian conditioning,” in Contemporary Learning Theories: Instrumental Conditioning Theory and the Impact of Biological Constraints on Learning, eds Klein S. B. Mowrer R. R. (Hillsdale, NJ: Erlbaum), 237–275

[B50] Garraway S. M. Mendell L. M. (2007). Spinal cord transection enhances afferent-evoked inhibition in lamina II neurons and abolishes BDNF-induced facilitation of their sensory input. J. Neurotrauma 24, 379–390 10.1089/neu.2006.0115 17376001

[B51] Garraway S. M. Petruska J. C. Mendell L. M. (2003). BDNF sensitizes the response of lamina II neurons to high threshold primary afferent inputs. Eur. J. Neurosci. 18, 2467–2476 10.1046/j.1460-9568.2003.02982.x 14622147

[B52] Garraway S. M. Turtle J. D. Huie J. R. Lee K. H. Hook M. A. Woller S. A. Grau J. W. (2011). Intermittent noxious stimulation following spinal cord contusion injury impairs locomotor recovery and reduces spinal BDNF-TrkB signaling in adult rats. Neuroscience 199, 86–102 10.1016/j.neuroscience.2011.10.007 22027236 PMC3237800

[B53] Gibson J. (1979). The Ecological Approach to Visual Perception. Boston: Houghton Mifflin

[B54] Gjerstad J. Tjolsen A. Hole K. (2001). Induction of long-term potentiation of single wide dynamic range neurones in the dorsal horn is inhibited by descending pathways. Pain 91, 263–268 10.1016/S0304-3959(00)00448-6 11275383

[B55] Gómez-Pinilla F. Huie J. R. Ying Z. Ferguson A. Crown E. D. Baumbauer K. M. Edgerton V. R. Grau J. W. (2007). BDNF and learning: evidence that instrumental training promotes learning within the spinal cord by up-regulating BDNF expression. Neuroscience 148, 893–906 10.1016/j.neuroscience.2007.05.051 17719180 PMC3225191

[B56] Grau J. W. (2010). “Instrumental conditioning,” in Corsini Encyclopedia of Psychology, 4th Edn, eds Weiner I. B. Nemeroff C. B. (New York: John Wiley & Sons), 480–481

[B57] Grau J. W. Barstow D. G. Joynes R. L. (1998). Instrumental learning within the spinal cord: I. Behavioral properties. Behav. Neurosci. 112, 1366–1386 10.1037/0735-7044.112.6.1366 9926819

[B58] Grau J. W. Crown E. D. Ferguson A. R. Washburn S. N. Hook M. A. Miranda R. C. (2006). Instrumental learning within the spinal cord: underlying mechanisms and implications for recovery after injury. Behav. Cogn. Neurosci. Rev. 5, 191–239 10.1177/1534582306289738 17099112

[B59] Grau J. W. Joynes R. L. (2005a). A neural-functionalist approach to learning. Int. J. Comp. Psychol. 18, 1–22

[B60] Grau J. W. Joynes R. L. (2005b). Neurofunctionalism revisited: learning is more than you think it is. Int. J. Comp. Psychol. 18, 46–59

[B61] Grau J. W. Salinas J. A. Illich P. A. Meagher M. W. (1990). Associative learning and memory for an antinociceptive response in the spinalized rat. Behav. Neurosci. 104, 489–494 10.1037/0735-7044.104.3.489 2162184

[B62] Grau J. W. Washburn S. N. Hook M. A. Ferguson A. R. Crown E. D. Garcia G. Bolding K. A. Miranda R. C. (2004). Uncontrollable nociceptive stimulation undermines recovery after spinal cord injury. J. Neurotrauma 21, 1795–1817 10.1089/neu.2004.21.1795 15684770

[B63] Grillner S. Wallen P. (1985). Central pattern generators for locomotion, with special reference to vertebrates. Ann. Rev. Neurosci. 8, 233–261 10.1146/annurev.ne.08.030185.001313 2984978

[B64] Groves P. M. Thompson R. F. (1970). Habituation: a dual-process theory. Psychol. Rev. 77, 419–450 10.1037/h0029810 4319167

[B65] Gwak Y. S. Hulsebosch C. E. (2011). GABA and central neuropathic pain following spinal cord injury. Neuropharmacology 60, 799–808 10.1016/j.neuropharm.2010.12.030 21216257 PMC3285561

[B66] Hains B. C. Everhart A. W. Fullwood S. D. Hulsebosch C. E. (2002). Changes in serotonin, serotonin transporter expression and serotonin denervation supersensitivity: involvement in chronic central pain after spinal hemisection in the rat. Exp. Neurol. 175, 347–362 10.1006/exnr.2002.7892 12061865

[B67] Harkema S. Gerasimenko Y. Hodes J. Burdick J. Angeli C. Chen Y. Ferreira C. Willhite A. Rejc E. Grossman R. G. Edgerton V. R. (2011). Effect of epidural stimulation of the lumbosacral spinal cord on voluntary movement, standing, and assisted stepping after motor complete paraplegia: a case study. Lancet 377, 1938–1947 10.1016/S0140-6736(11)60547-3 21601270 PMC3154251

[B68] Heppenstall P. A. Lewin G. R. (2001). BDNF but not NT-4 is required for normal flexion reflex plasticity and function. Proc. Natl. Acad. Sci. U.S.A. 98, 8107–8112 10.1073/pnas.141015098 11438749 PMC35475

[B69] Herrero J. F. Laird J. M. Lopez-Garcia J. A. (2000). Wind-up of spinal cord neurones and pain sensation: much ado about something? Prog. Neurobiol. 61, 169–203 10.1016/S0301-0082(99)00051-9 10704997

[B70] Hillgard E. R. Marquis D. G. (1940). Conditioning and Learning. New York: Appleton

[B71] Hook M. A. Grau J. W. (2007). An animal model of functional electrical stimulation: evidence that the central nervous system modulates the consequences of training. Spinal Cord 45, 702–712 10.1038/sj.sc.3102096 17700514 PMC3222458

[B72] Hook M. A. Huie J. R. Grau J. W. (2008). Peripheral inflammation undermines the plasticity of the isolated spinal cord. Behav. Neurosci. 122, 233–249 10.1037/0735-7044.122.1.233 18298266 PMC2665167

[B73] Horridge G. A. (1962). Learning of the leg position by the ventral nerve cord in headless insects. Proc. R. Soc. Lond. B Biol. Sci. 157, 33–52 10.1098/rspb.1962.0061

[B74] Huie J. R. Baumbauer K. M. Lee K. H. Beattie M. S. Bresnahan J. C. Ferguson A. R. Grau J. W. (2012a). Glial tumor necrosis factor alpha (TNFα) generates metaplastic inhibition of spinal learning. PLoS ONE 7, e39751 10.1371/journal.pone.0039751 22745823 PMC3379985

[B75] Huie J. R. Garraway S. M. Hoy K. C. Grau J. W. (2012b). Learning in the spinal cord: BDNF mediates the beneficial effects of instrumental training. Neuroscience 200, 74–90 10.1016/j.neuroscience.2011.10.028 22056599 PMC3249495

[B76] Illich P. A. Salinas J. A. Grau J. W. (1994). Latent inhibition and overshadowing of an antinociceptive response in spinalized rats. Behav. Neural Biol. 62, 140–150 10.1016/S0163-1047(05)80035-4 7993304

[B77] Ji R.-R. Kohno T. Moore K. A. Woolf C. J. (2003). Central sensitization and LTP: do pain and memory share similar mechanisms. Trends Neurosci. 26, 696–705 10.1016/j.tins.2003.09.017 14624855

[B78] Joynes R. L. Ferguson A. R. Crown E. D. Patton B. C. Grau J. W. (2003). Instrumental learning within the spinal cord: V. Evidence the behavioral deficit observed after noncontingent nociceptive stimulation reflects an intraspinal modification. Behav. Brain Res. 141, 159–170 10.1016/S0166-4328(02)00372-8 12742252

[B79] Joynes R. L. Grau J. W. (1996). Mechanisms of Pavlovian conditioning: the role of protection from habituation in spinal conditioning. Behav. Neurosci. 110, 1375–1387 10.1037/0735-7044.110.6.1375 8986339

[B80] Joynes R. L. Grau J. W. (2004). Instrumental learning within the spinal cord: III. Prior exposure to noncontingent shock induces a behavioral deficit that is blocked by an opioid antagonist. Neurobiol. Learn. Mem. 82, 35–51 10.1016/j.nlm.2004.04.001 15183169

[B81] Joynes R. L. Janjua K. R. Grau J. W. (2004). Instrumental learning within the spinal cord: VI. Disruption of learning by the NMDA antagonist APV. Behav. Brain Res. 154, 431–438 10.1016/j.bbr.2004.03.030 15313031

[B82] Kandel E. R. Schwartz J. H. (1982). Molecular biology of learning: modification of transmitter release. Science 218, 433–442 10.1126/science.6289442 6289442

[B83] Kang H. Schuman E. M. (1995). Long-lasting neurotrophin-induced en- hancement of synaptic transmission in the adult hippocampus. Science 267, 1658–1662 10.1126/science.7886457 7886457

[B84] Kerr B. J. Bradbury E. J. Bennett D. L. H. Trivedi P. M. Dassan P. French J. Shelton D. B. McMahon S. B. Thompson S. W. N. (1999). Brain-derived neurotrophic factor modulates nociceptive sensory inputs and NMDA-evoked responses in the rat spinal cord. J. Neurosci. 19, 5138–5148 10366647 10.1523/JNEUROSCI.19-12-05138.1999 PMC6782650

[B85] Konorski J. A. (1948). Conditioned Reflexes and Neuron Organization. New York: Hafner Publishing Company

[B86] Konorski J. A. Miller S. M. (1937). On two types of conditioned reflex. J. Gen. Psychol. 16, 264–273 10.1080/00221309.1937.9917950

[B87] Latremoliere A. Woolf C. J. (2009). Central sensitization: a generator of pain hypersensitivity by central neural plasticity. J. Pain. 10, 895–926 10.1016/j.jpain.2009.06.012 19712899 PMC2750819

[B88] Lee R. Kermani P. Teng K. K. Hempstead B. L. (2001). Regulation of cell survival by secreted neurotrophins. Science 294, 1945–1948 10.1126/science.1065329 11729324

[B89] Lever I. Cunningham J. Grist J. Yip P. K. Malcangio M. (2003). Release of BDNF and GABA in the dorsal horn of neuropathic rats. Eur. J. Neurosci. 18, 1169–1174 10.1046/j.1460-9568.2003.02848.x 12956715

[B90] Liu G. T. Crown E. D. Miranda R. C. Grau J. W. (2005). Instrumental learning within the rat spinal cord: localization of the essential neural circuit. Behav. Neurosci. 119, 538–547 10.1037/0735-7044.119.2.538 15839800

[B91] Lu B. Pang P. T. Woo N. H. (2005). The yin and yang of neurotrophin action. Nat. Rev. Neurosci. 6, 603–614 10.1038/nrn1726 16062169

[B92] Lu V. B. Biggs J. E. Stebbing M. J. Balasubramanyan S. Todd K. G. Lai A. Y. Colmers W. F. Dawbarn D. Ballanyi K. Smith P. A. (2009). Brian-derived neurotrophic factor drives the changes in excitatory synaptic transmission in the rat superficial dorsal horn that follow sciatic nerve injury. J. Physiol. (Lond.) 587, 1013–1032 10.1113/jphysiol.2008.166306 19124536 PMC2673772

[B93] Lu Y. Ji Y. Ganesan S. Schlosser R. Martinowich K. Sun M. Mei F. Chao M. V. Lu B. (2011). TrkB as a potential synaptic and behavioral tag. J. Neurosci. 31, 11762–11771 10.1523/JNEUROSCI.2364-11.2011 21849537 PMC3169103

[B94] Ma Q. P. Woolf C. J. (1995). Noxious stimuli induce an N-methyl-D-aspartate receptor-dependent hypersensitivity of the flexion withdrawal reflex to touch: implications for the treatment of mechanical allodynia. Pain 61, 383–390 10.1016/0304-3959(94)00195-K 7478681

[B95] Maier S. F. Seligman M. E. P. (1976). Learned helplessness: theory and evidence. J. Exp. Psychol. Gen. 105, 3–46 10.1037/0096-3445.105.1.3

[B96] Meagher M. W. Chen P. Salinas J. A. Grau J. W. (1993). Activation of the opioid and nonopioid hypoalgesic systems at the level of the brainstem and spinal cord: does a coulometric relation predict the emergence or form of environmentally-induced hypoalgesia? Behav. Neurosci. 107, 493–505 10.1037/0735-7044.107.3.493 8392349

[B97] Melzack K. Wall P D. (1965). Pain mechanisms: a new theory. Science 150, 971–979 10.1126/science.150.3699.971 5320816

[B98] Mendell L. M. (1966). Physiological properties of unmyelinated fibre projection to the spinal cord. Exp. Neurol. 16, 316–333 10.1016/0014-4886(66)90068-9 5928985

[B99] Merighi A. Salio C. Ghirri A. Lossi L. Ferrini F. Betelli C. Bardoni R. (2008). BDNF as a pain modulator. Prog. Neurobiol. 85, 297–317 10.1016/j.pneurobio.2008.04.004 18514997

[B100] Miki K. Fukuoka T. Tokunaga A. Kondo E. Dai Y. Noguchi K. (2000). Differential effect of brain-derived neurotrophic factor on high-threshold mechanosensitivity in a rat neuropathic pain model. Neurosci. Lett. 278, 85–88 10.1016/S0304-3940(99)00908-8 10643807

[B101] Millan M. J. (2002). Descending control of pain. Prog. Neurobiol. 66, 355–474 10.1016/S0301-0082(02)00009-6 12034378

[B102] Morris R. G. M. (1994). “The neural basis of learning with particular reference to the role of synaptic plasticity,” in Animal Learning and Cognition, ed. Mackintosh N. J. (New York: Academic Press), 135–184

[B103] Morris R. G. M. Anderson E. Lynch G. S. Baudry M. (1986). Selective impairment of learning and blockade of long-term potentiation by an N-methyl-d-aspartate receptor antagonist, AP5. Nature 319, 774–776 10.1038/319696b0 2869411

[B104] Moser E. I. Moser M. B. (1999). Is learning blocked by saturation of synaptic weights in the hippocampus? Neurosci. Biobehav. Rev. 23, 661–672 10.1016/S0149-7634(98)00060-8 10392658

[B105] Nishimaru H. Kudo N. (2000). Formation of the central pattern generator for locomotion in the rat and mouse. Brain Res. Bull. 53, 661–669 10.1016/S0361-9230(00)00399-3 11165801

[B106] Patterson M. M. (2001). “Classical conditioning of spinal reflexes: the first seventy years,” in Model Systems and the Neurobiology of Associative Learning: A Festschrift in Honor of Richard F. Thompson, eds Steinmetz J. E. Gluck M. A. Solomon P. R. (Mahwah, NJ: Erlbaum), 1–22

[B107] Patterson M. M. Cegavske C. F. Thompson RF. (1973). Effects of a classical conditioning paradigm on hind-limb flexion nerve response in the immobilized cat. J. Comp. Physiol. Psychol. 84, 88–97 10.1037/h0035021 4717554

[B108] Patterson M. M. Grau J. W. (2001). Spinal Cord Plasticity: Alterations in Reflex Function. Norwell, MA: Kluwer Academic Publishers

[B109] Patterson S. L. Abel T. Deuel T. A. Martin K. C. Rose J. C. Kandel E. R. (1996). Recombinant BDNF rescues deficits in basal synaptic transmission and hippocampal LTP in BDNF knockout mice. Neuron 16, 1137–1145 10.1016/S0896-6273(00)80140-3 8663990

[B110] Patton B. C. Hook M. A. Crown E. D. Ferguson A. R. Grau J. W. (2004). The behavioral deficit observed following noncontingent shock in spinalized rats is prevented by the protein synthesis inhibitor cycloheximide. Behav. Neurosci. 118, 653–658 10.1037/0735-7044.118.3.653 15174945

[B111] Pezet S. Malcangio M. Lever I. J. Perkinton M. S. Thompson S. W. Williams R. J. McMahon S. B. (2002). Noxious stimulation induces Trk receptor and downstream ERK phosphorylation in spinal dorsal horn. Mol. Cell. Neurosci. 21, 684–695 10.1006/mcne.2002.1205 12504600

[B112] Pittenger C. Kandel R. R. (2003). In search of general mechanisms of long-lasting plasticity: aplysia and the hippocampus. Philos. Trans. R. Soc. Lond. B Biol. Sci. 358, 757–763 10.1098/rstb.2002.1247 12740123 PMC1693156

[B113] Rescorla R. A. (1988). Behavioral studies of Pavlovian conditioning. Ann. Rev. Neurosci. 11, 329–352 10.1146/annurev.ne.11.030188.001553 3284445

[B114] Sahley C. Crow T. (1998). “Invertebrate learning: current Perspectives,” in Neurobiology of Learning & Memory, eds Martinez J. Kesner R. (San Diego, CA: Academic Press), 177–203

[B115] Sandkühler J. (2000). Learning and memory in pain pathways. Pain 88, 113–118 10.1016/S0304-3959(00)00424-3 11050365

[B116] Sandkühler J. Liu X. (1998). Induction of long-term potentiation at spinal synapses by noxious stimulation or nerve injury. Eur. J. Neurosci. 10, 2476–2480 10.1046/j.1460-9568.1998.00278.x 9749775

[B117] Skinner B. F. (1938). The Behavior of Organisms. Englewood Cliffs, NJ: Prentice-Hall

[B118] Staubli U. Thibault O. DiLorenzo M. Lynch G. (1989). Antagonism of NMDA receptors impairs acquisition but not retention of olfactory memory. Behav. Neurosci. 103, 54–60 10.1037/0735-7044.103.1.54 2564277

[B119] Timberlake W. (1999). “Biological behaviorism,” in Handbook of Behaviorism, eds O’Donohue W. Kitchener R. (San Diego, CA: Academic Press), 243–284

[B120] Timberlake W. Lucas G. A. (1989). “Behavior systems and learning: from misbehavior to general principles,” in Contemporary Learning Theories: Instrumental Conditioning and the Impact of Biological Constraints on Learning, eds Klein S. B. Mowrer R. R. (Hillsdale, NJ: Erlbaum), 237–275

[B121] Vichaya E. G. Baumbauer K. M. Carcoba L. M. Grau J. W. Meagher M. W. (2009). Spinal glia modulate both adaptive and pathological processes. Brain Behav. Immun. 23, 969–976 10.1016/j.bbi.2009.05.001 19435601 PMC2749915

[B122] Washburn S. N. Maultsby M. L. Puga D. A. Grau J. W. (2008). Opioid regulation of spinal cord plasticity: evidence the kappa-2 opioid receptor agonist GR89686 inhibits learning within the rat spinal cord. Neurobiol. Learn. Mem. 89, 1–16 10.1016/j.nlm.2007.09.009 17983769 PMC2171051

[B123] Washburn S. N. Patton B. C. Ferguson A. R. Hudson K. L. Grau J. W. (2007). Exposure to intermittent nociceptive stimulation under pentobarbital anesthesia disrupts spinal cord function in rats. Psychopharmacology 192, 243–252 10.1007/s00213-007-0707-1 17297638 PMC3222461

[B124] Watson C. Paxinos G. Kayalioglu G. (2008). The Spinal Cord: A Christopher and Dana Reeve Foundation Text and Atlas. San Diego, CA: Academic Press

[B125] Willis W. D. (2001). “Mechanisms of central sensitization of nociceptive dorsal horn neurons,” in Spinal Cord Plasticity: Alterations in Reflex Function, eds Patterson M. M. Grau J. W. (Norwell, MA: Kluwer Academic Publishers), 127–162

[B126] Windhorst U. (2007). Muscle proprioceptive feedback and spinal networks. Brain Res. Bull. 73, 155–202 10.1016/j.brainresbull.2007.03.010 17562384

[B127] Woolf C. J. (1983). Evidence for a central component of post-injury pain hypersensitivity. Nature 305, 686–688 10.1038/306686a0 6656869

[B128] Woolf C. J. Thompson S. W. N. (1991). The induction and maintenance of central sensitization is dependent on N-methyl-D-aspartic acid receptor activation; implications for the treatment of post-injury pain hypersensitivity states. Pain 44, 293–299 10.1016/0304-3959(91)90100-C 1828878

[B129] Young E. E. Baumbauer K. M. Elliot A. Joynes R. L. (2007). Lipopolysaccharide induces a spinal learning deficit that is blocked by IL-1 receptor antagonism. Brain Behav. Immun. 21, 748–757 10.1016/j.bbi.2007.02.001 17382514

[B130] Zhao J. Seereeram A. Nassar M. A. Levato A. Pezet S. Hathaway G. Morenilla-Palao C. Stirling C. Fitzgerald M. McMahon S. B. Rios M. Wood J. N. London Pain Consortium (2006). Nociceptor-derived brain-derived neurotrophic factor regulates acute and inflammatory but not neuropathic pain. Mol. Cell. Neurosci. 31, 539–548 10.1016/j.mcn.2005.11.008 16413788

[B131] Zhou L.-J. Zhong Y. Ren W.-J. Li Y.-Y. Zhang T. Liu X.-G. (2008). BDNF induces late-phase LTP of C-fiber evoked field potentials in rat spinal dorsal horn. Exp. Neurol. 212, 507–514 10.1016/j.expneurol.2008.04.034 18565512

